# A compendium of multi-omics data illuminating host responses to lethal human virus infections

**DOI:** 10.1038/s41597-024-03124-3

**Published:** 2024-04-02

**Authors:** Amie J. Eisfeld, Lindsey N. Anderson, Shufang Fan, Kevin B. Walters, Peter J. Halfmann, Danielle Westhoff Smith, Larissa B. Thackray, Qing Tan, Amy C. Sims, Vineet D. Menachery, Alexandra Schäfer, Timothy P. Sheahan, Adam S. Cockrell, Kelly G. Stratton, Bobbie-Jo M. Webb-Robertson, Jennifer E. Kyle, Kristin E. Burnum-Johnson, Young-Mo Kim, Carrie D. Nicora, Zuleyma Peralta, Alhaji U. N’jai, Foday Sahr, Harm van Bakel, Michael S. Diamond, Ralph S. Baric, Thomas O. Metz, Richard D. Smith, Yoshihiro Kawaoka, Katrina M. Waters

**Affiliations:** 1 Department of Pathobiological Sciences, University of Wisconsin—Madison, Madison, WI 53706 USA; 2 Biological Sciences Division, Earth and Biological Sciences Directorate, Pacific Northwest National Laboratory, Richland, WA 99352 USA; 3 Department of Medicine, Washington University School of Medicine, Saint Louis, MO 63110 USA; 4 Department of Epidemiology, University of North Carolina at Chapel Hill, North Carolina, 27599 USA; 5 Department of Microbiology and Immunology, University of North Carolina, Chapel Hill, NC 27599 USA; 6 Department of Genetics and Genomic Sciences, Icahn School of Medicine at Mount Sinai, New York City, NY 10029 USA; 7 Department of Biological Sciences, Fourah Bay College, Freetown, Sierra Leone; 8 Department of Microbiology, College of Medicine and Allied Health Sciences, University of Sierra Leone, Freetown, Sierra Leone; 9 Department of Medical Education, California University of Science and Medicine, Colton, CA 92324 USA; 10 Department of Microbiology, College of Medicine and Health Sciences, University of Sierra Leone, Freetown, Sierra Leone; 11 Icahn Genomics Institute, Icahn School of Medicine at Mount Sinai, New York City, NY 10029 USA; 12 Department of Microbiology, Icahn School of Medicine at Mount Sinai, New York City, NY 10029 USA; 13 Department of Pathology and Immunology, Washington University School of Medicine, Saint Louis, MO 63110 USA; 14 Department of Molecular Microbiology, Washington University School of Medicine, Saint Louis, MO 63110 USA; 15 Department of Microbiology and Immunology, Institute of Medical Science, University of Tokyo, 108-8639 Tokyo, Japan; 16 The Research Center for Global Viral Diseases, National Center for Global Health and Medicine Research Institute, Tokyo, 108-8639 Japan; 17 Present Address: Coronavirus and Other Respiratory Viruses Laboratory Branch (CRVLB), Coronavirus and Other Respiratory Viruses Division (CORVD), National Center for Immunization and Respiratory Diseases (NCIRD), Centers for Disease Control and Prevention (CDC), Atlanta, GA 30329 USA; 18 Present Address: Integrated Research Facility at Fort Detrick, National Institute of Allergy and Infectious Diseases, National Institutes of Health, Frederick, MD 21702 USA; 19 Present Address: Department of Surgery, University of Minnesota, Minneapolis, MN 55455 USA; 20 Present Address: Nuclear, Chemistry, and Biosciences Division; National Security Directorate, Pacific Northwest National Laboratory, Richland, WA 99352 USA; 21 Present Address: Department of Microbiology and Immunology, University of Texas Medical Branch, Galveston, TX 77555 USA; 22 Present Address: Solid Biosciences, Charlston, MA 02139 USA; 23 Present Address: Partillion Bioscience, Los Angeles, CA 90064 USA

**Keywords:** Data publication and archiving, Viral infection

## Abstract

Human infections caused by viral pathogens trigger a complex gamut of host responses that limit disease, resolve infection, generate immunity, and contribute to severe disease or death. Here, we present experimental methods and multi-omics data capture approaches representing the global host response to infection generated from 45 individual experiments involving human viruses from the *Orthomyxoviridae*, *Filoviridae*, *Flaviviridae*, and *Coronaviridae* families. Analogous experimental designs were implemented across human or mouse host model systems, longitudinal samples were collected over defined time courses, and global multi-omics data (transcriptomics, proteomics, metabolomics, and lipidomics) were acquired by microarray, RNA sequencing, or mass spectrometry analyses. For comparison, we have included transcriptomics datasets from cells treated with type I and type II human interferon. Raw multi-omics data and metadata were deposited in public repositories, and we provide a central location linking the raw data with experimental metadata and ready-to-use, quality-controlled, statistically processed multi-omics datasets not previously available in any public repository. This compendium of infection-induced host response data for reuse will be useful for those endeavouring to understand viral disease pathophysiology and network biology.

## Background & Summary

**Fig. 1:**
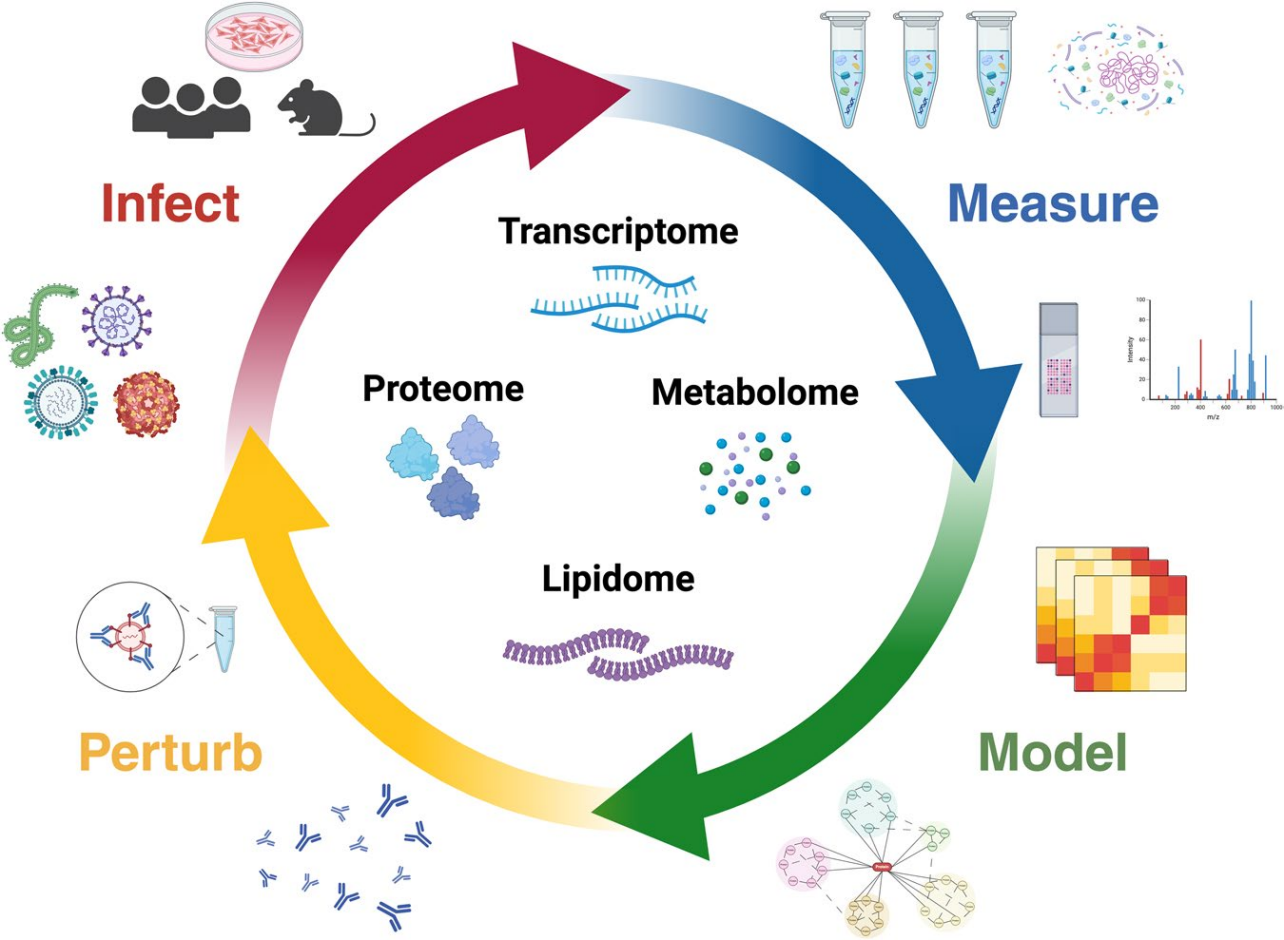
The OMICS-LHV project used the systems biology paradigm to evaluate host responses to lethal virus infections. Samples were obtained from human or mouse cells and tissues infected with different viruses, host responses were measured using multi-omics approaches, and data were statistically processed using provided in-house developed software. From this, models of host responses may be used to develop hypotheses, which may be tested by perturbing the system and repeating the systems biology data analysis lifecycle.

**Fig. 2:**
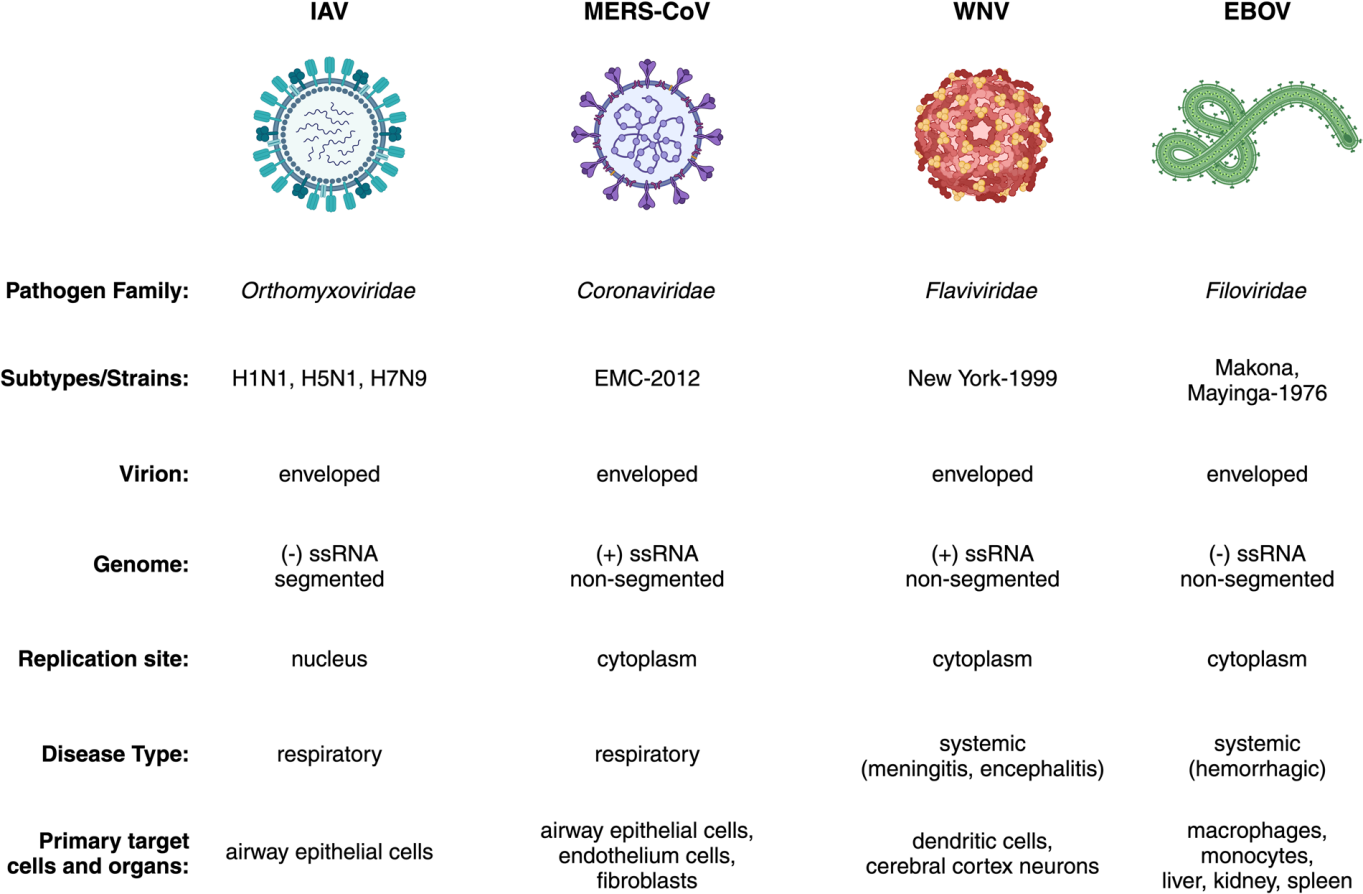
The OMICS-LHV project focused on Influenza A virus (IAV), Middle Eastern Respiratory Syndrome-related Coronavirus (MERS-CoV), West Nile virus (WNV), and Ebola virus (EBOV). The figure summarizes basic virology information covering pathogenic family, strains or subtypes used, virion type, genomic organization, replication site, disease type, and primary target cells and/or organs used in experimental designs. The virus strains used in the reported studies are provided, with additional details provided in the Methods and Supplementary Table 1.

The ‘Omics of Lethal Human Viruses (OMICS-LHV) Systems Biology Center was funded by the National Institutes of Allergy and Infectious Diseases (NIAID) from June 2013 to June 2018 (grant # U19AI106772), and was tasked with using a systems biology approach (Fig. 1) to study host responses to four viral pathogens that cause lethal disease in humans: Influenza A virus (IAV, *Orthomyxoviridae family*), Ebola virus (EBOV, *Filoviridae family*), West Nile virus (WNV, *Flaviviridae family*), and Middle East respiratory syndrome coronavirus (MERS-CoV, *Coronaviridae family*) (an overview of basic virology and virus-associated diseases are provided in Fig. 2). These viruses comprise some of the most lethal and debilitating pathogens known to humans, exhibit significant potential for emergence of new pandemic strains, and impose substantial public health and economic burdens on the world community. As such, they are classified as Category A (EBOV), B (WNV), or C (IAV and MERS-CoV) priority pathogens by the NIAID^1^. Host responses against all four viruses are thought to contribute to pathogenesis in severe and fatal cases^2,3^. Therefore, the overarching goal of the OMICS-LHV Systems Biology Center was to use global host response data to model virus infections and identify host-dependent mechanisms that regulate severe or fatal disease.

The work performed by the OMICS-LHV Systems Biology Center built upon previous systems biology studies of influenza A viruses (moderate pathogenicity 2009 pandemic H1N1 [pH1N1] and highly pathogenic H5N1 avian influenza viruses) and severe acute respiratory syndrome coronavirus (SARS-CoV), which consisted primarily of transcriptomics (mRNA) and proteomics analyses of global host responses in human respiratory epithelial cells and mouse lung tissue^4^. The OMICS-LHV Center expanded upon this work in several ways: (***i***) Studies of host responses to pH1N1 and H5N1 virus infections were extended to include a full panel of multi-omics analyses, including transcriptomics (mRNA and microRNA), proteomics, metabolomics, and lipidomics; (***ii***) Host responses to newly emerging human respiratory pathogens (H7N9 influenza virus and MERS-CoV) and other important non-respiratory pathogens (WNV and Ebola virus) also were examined by multi-omics analysis; (***iii***) In most cases, host responses were measured in multiple cell or tissue types after infection with a particular virus or set of viruses; and (***iv***) For Ebola virus, host responses were determined in blood components (*i.e*., plasma or peripheral blood mononuclear cells [PBMC]) of naturally infected humans.

## Experimental Design Overview

Herein, we report 45 unique experiments carried out by the OMICS-LHV Systems Biology Center. We define an experiment as comprising the infection or interferon treatment of cells or mice, followed by sample collection over a time course, and subsequent multi-omics analysis of the collected samples. In many experiments, samples were collected in parallel to allow for transcriptomics, proteomics, metabolomics, and lipidomics analyses. In other experiments, samples were collected for analysis of a subset of the available omics platforms. For all experiments, we assigned a unique experiment identifier to facilitate communication and to enable integration of multi-omics datasets derived from the same sample collection experiment. For each virus family, a panel of viruses that included wild-type strains and mutants was used for infection experiments and multi-omics data collection (see Supplementary Table 1, for a list of viruses used in the studies described herein).

**Fig. 3:**
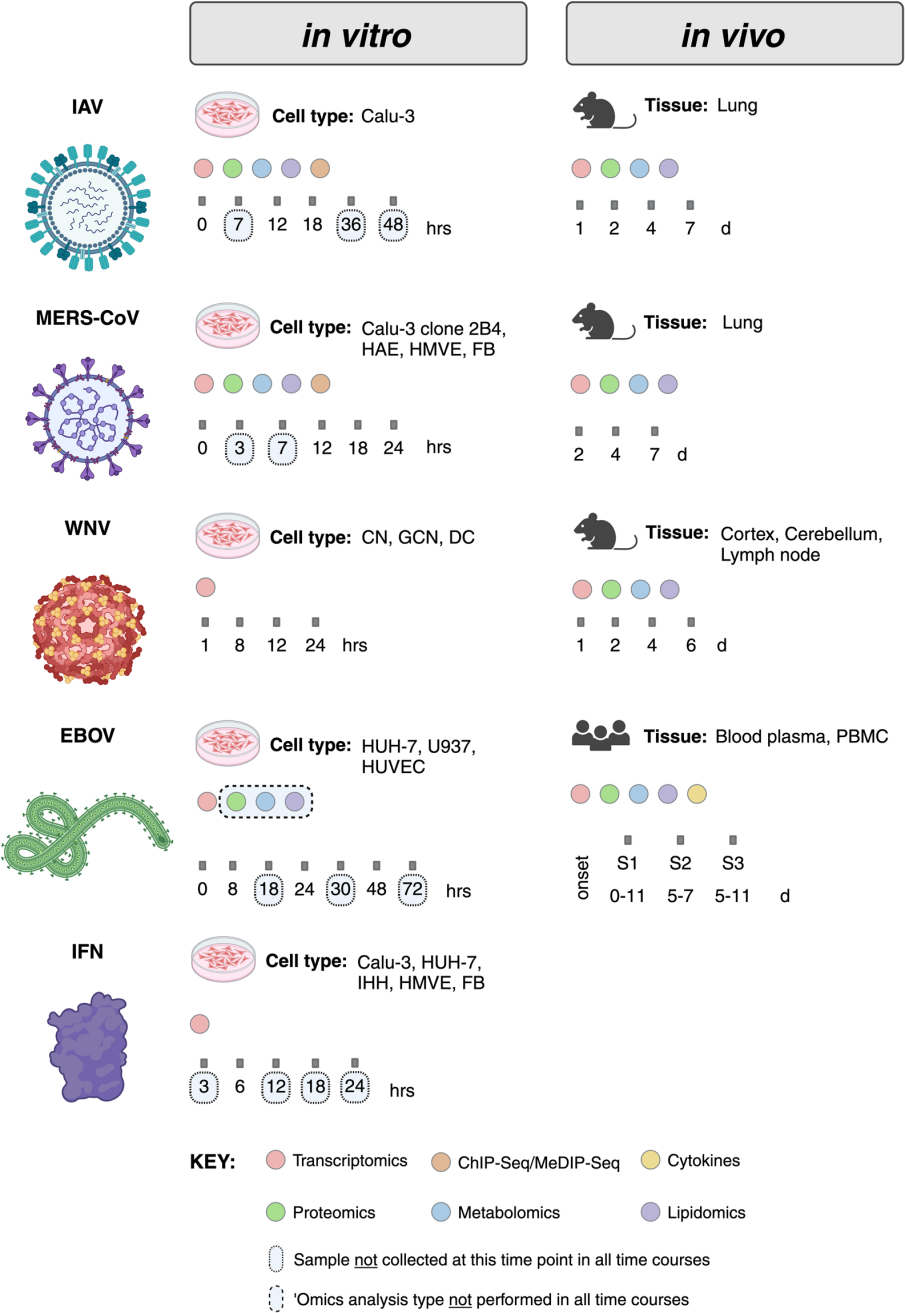
Model systems and timelines are summarized for multi-omics sample collection experiments *in vitro* and *in vivo*. For experimental design comparisons across the representative viral studies (IAV, MERS-CoV, WNV, and EBOVs), we have provided experimental conditions, including the cell or tissue type analysed, time points at which samples were obtained, and the omics data type collected. In all cell- or mouse-based model system experiments, transcriptomics analyses were done using mRNA or microRNA microarrays; while in the study of humans naturally infected with Ebola virus, transcriptomics analyses were done using RNA-Seq. ChIP-Seq and MeDIP-Seq experiments were carried out in Calu-3 cells (IAV) or Calu-3/2B4 cells (MERS-CoV) only. S1, S2, and S3 refer to blood sample 1, sample 2, and sample 3, respectively, collected from human patients infected with Ebola virus.

All experiments were designed with input and collaboration between experimental, technical, and computational scientists and relied on extensive previous work using omics-based approaches to study host responses to viral infections^4^. The overall goal was to collect samples from various *in vitro* and *in vivo* infection models and perform multi-omics analyses comprising global transcriptomics (mRNA and microRNA), proteomics, metabolomics, and lipidomics; however, in some experiments only transcriptomics analyses were performed (Fig. 3). A detailed overview experimental model systems (including cell and tissue types, see Supplementary Table 5 for cell line acronyms and definitions), infection/treatment conditions, longitudinal sample collection time points, and omics analyses performed have been provided in Supplementary Table 2.). For most experiments, transcriptomics was assessed by using mRNA or microRNA microarrays, while transcriptomics of host responses to natural Ebola virus infection in humans was determined by using RNA sequencing (RNA-Seq). Proteomics, metabolomics, and lipidomics were assessed using mass spectrometry-based approaches. Analysis of mRNA and microRNAs was done with the same total RNA extract; and analyses of proteins, metabolites, and lipids were carried out with extracts prepared simultaneously from the same sample. The only exception to the latter is human plasma, for which highly abundant proteins were depleted prior to preparing the protein extract. In one set of experiments, epigenetic changes were investigated by chromatin immunoprecipitation sequencing (ChIP-Seq) or methylated DNA immunoprecipitation sequencing (MeDIP-Seq). When multi-omics analysis was performed and multiple extraction methods were required, whenever possible, parallel samples were collected in the same experiment. An overview of each experiment sample type (cell culture, tissue, mouse, or human) and specific methodological details for each are provided in the Methods section.

### Cell culture experiments

Infections or treatments were carried out in cell lines or primary cells representing cell types targeted by viruses during natural infection and are known to be permissive to infection with a given virus *in vitro*. For infection experiments, the multiplicity of infection (*i.e*., the number of infectious virus particles given per cell) used varied by experiment, but the overall goal was to achieve uniform infection of most cells at the time of inoculation. Sample collection time points are well-aligned across the different virus infection models (Fig. 3), and were selected to allow for analysis of host responses from the time of infection through the destruction of the cell monolayer. Similar infection timelines in previous experiments allowed for the identification of clear patterns of host gene expression^4–11^. For interferon treatment experiments, cells were treated with recombinant type I or type II interferon proteins and samples were collected at a subset of the time points used for infection experiments. Mock controls comprised cells that were treated exactly as described for infection or interferon exposure, except without the addition of infectious virus or recombinant interferon proteins, and samples were collected at the same time points as for infected or treated cells. In all experiments, 3–6 replicate samples were collected for each omics analysis type in each infection/treatment and time point condition.

### Mouse experiments

All mice used are susceptible to infection with the indicated viruses. For IAV and WNV, infections were carried out in wild-type C57BL/6 J mice, and infections with MERS-CoV were performed in C57BL/6 J mice expressing the human dipeptidyl peptidase 4 (DPP4) gene (C57BL/6J-hDPP4)^12^. The sex and age of the mice varied depending on the virus used and were selected to align with established precedents. Mice of the appropriate age and sex (either purchased from a vendor or derived from internal breeding colonies) were grouped randomly for infection experiments. Virus dosages and routes of inoculation also varied by the virus and/or target tissue: in all cases, the selected dosages are known to cause substantial disease and the selected routes of inoculation are consistent with established protocols for the study of each virus. Mock-infected controls comprised mice inoculated with PBS (no virus), and tissues from mock-infected control mice were collected at the same time points as for infected mice. Sample collection time points focus primarily on the early stage of virus-induced disease and, in general, are well-aligned across the different virus infection models (Fig. 3). Only relevant target tissues for each virus were collected for multi-omics analyses (*i.e*., lungs for influenza and MERS-CoV; and brain, lymph nodes, or serum for WNV). In all experiments, 3–10 replicate samples were collected for each analysis type in each infection and time point condition.

### Human study

For individuals naturally infected with Ebola virus, blood samples were collected at the time of admission to an Ebola Treatment Center and, if possible, at additional time points over their infection and recovery^13^. Peripheral blood mononuclear cells (PBMCs) and plasma were isolated from whole blood. PBMCs were used for transcriptomics analysis and plasma was used for proteomics, metabolomics, and lipidomics analyses. Samples were collected from 20 Ebola virus-positive patients (11 survivors and 9 fatalities, with multiple samples from survivors) and 10 healthy volunteers (Fig. 3). Upon recruitment, basic demographic criteria (*i.e*., age, sex) and clinical (*i.e*., time since symptom onset, stage of disease) information was collected^13^. In addition, the viral load was determined by qRT-PCR analysis of total RNA extracted from PBMCs and inflammatory cytokine concentrations were measured in the plasma^13^.

## Methods

### Ethics statements

#### Animals

All animal experiments and procedures were approved by the Institutional Care and Use Committees of the University of Wisconsin (UW)-Madison School of Veterinary Medicine (protocol # V006426-A04), the University of North Carolina (UNC)-Chapel Hill (protocol # 16–251), or Washington University in St. Louis (WUSTL) (assurance number A3381-01) under relevant institutional and American Veterinary Association guidelines.

#### Humans

All work with samples from humans naturally infected with Ebola virus was approved by the UW-Madison Health Sciences Institutional Review Board (IRB) under protocol # 2015-0044. The protocol was also reviewed and approved by the Sierra Leone Ethics and Scientific Review Committee, the Research Ethics Review Committee of the Institute of Medical Science at the University of Tokyo, the IRBs of Icahn School of Medicine at Mount Sinai (ISMMS), and Pacific Northwest National Laboratory (PNNL). Ebola virus-positive patients were enrolled in the study after diagnosis and admission to Ebola Treatment Centers in Freetown, Sierra Leone in the months of February through May of 2015. Healthy subjects, who have had no prior exposure to Ebola virus disease, were recruited from healthcare workers and laboratory technicians during the same timeframe. Consent was obtained from all subjects by local medical staff prior to enrollment. For children under the age of 18, consent was provided by the child’s parent and/or legal guardian. All work related to resected human airway tissues was approved by the UNC-Chapel Hill IRB.

### Biosafety

The United States (US) Centers for Disease Control and Prevention (CDC) and/or the US Department of Agriculture approved the use of BSL-3, ABSL-3, ABSL-3+, and BSL-3Ag containment facilities at the UW-Madison, the UNC-Chapel Hill, and WUSTL.

#### Influenza viruses

All work with influenza viruses was performed at the UW-Madison. *In vitro* experiments with pH1N1 were performed in a biosafety level 2 (BSL-2) laboratory. *In vivo* experiments with pH1N1 and *in vitro* and *in vivo* experiments with H5N1 viruses were performed in an animal-enhanced biosafety level 3+ (ABSL-3+) laboratory. *In vitro* and *in vivo* experiments with H7N9 viruses were performed in a BSL-3-Agriculture (BSL-3Ag) laboratory.

#### Ebola viruses

All work with the biologically contained Ebola-ΔVP30 virus (see additional details below) was performed at the UW-Madison in BSL-2+ containment, under approval by the UW-Madison Institutional Biosafety Committee, the US CDC, and the US National Institutes of Health. Work with human samples containing authentic Ebola virus was performed in a field laboratory in Freetown, Sierra Leone. Prior to inactivation, samples were processed in portable, battery-operated, double HEPA-filtered, negative-pressure field laboratory containment units (*i.e*., Rapid Containment Kits, Germfree).

#### WNV

All *in vitro* and *in vivo* work with WNV was performed in BSL-3 or ABSL-3 laboratories at WUSTL.

#### MERS-CoV

All *in vitro* and *in vivo* work with MERS-CoV was performed in a BSL-3 laboratory at the UNC-Chapel Hill.

### Blinding and randomization

No blinding methods were used for collection of phenotypic data; however, it should be noted that most phenotypic data types collected herein (except haemorrhage scores in MERS-CoV-infected mice, see below) have quantitative outputs, which are less vulnerable to individual bias. Strategies for randomization are discussed in the section to which this information is appropriate, below.

### Experimental data documentation

For all experiments, primary metadata—that is, data related to the experimental design, collected phenotypic data, and the samples that were used for various analyses—were transcribed into documents with standardized formats. These primary metadata documents are publicly available and are described in more detail in the Data Records section.

### Virus strains

All wild-type and mutant viruses used in experiments comprising this collection are summarized in Supplementary Table 1. Requests to obtain virus(es) may be made by contacting the corresponding authors.

#### Influenza A viruses

Wild-type Influenza A virus (IAV) subtype strains included A/California/04/2009 (H1N1 subtype; ‘CA04’), A/Vietnam/1203/2004 (H5N1 subtype; ‘VN1203’), and A/Anhui/1/2013 (H7N9 subtype; ‘AH1’). CA04 and VN1203 were provided by the United States (US) Centers for Disease Control and Prevention (CDC) and AH1 was provided by Yuelong Shu (China CDC). VN1203 mutants include VN1203-PB2-627E (with a K → E amino acid substitution at PB2 residue 627)^14^ and VN1203-NS1trunc (with a stop codon at NS1 amino acid 124)^15^. AH1 mutants, which have not been described previously, include a virus possessing L103F and I106M amino acid substitutions in the NS1 protein (referred to as AH1-F/M) and a virus with several amino acid substitutions acquired during growth in the upper respiratory tract of ferrets (referred to as AH1-691). Wild-type and mutant VN1203 and AH1 viruses were rescued by reverse genetics as previously described^16–18^. Stock viruses were generated by passaging an aliquot of the original virus (CA04) or supernatants derived from reverse genetics transfection experiments (wild-type VN1203 or AH1 and their respective mutants) one time in MDCK cells, as previously described^19^, and stock virus titers were quantified by plaque assay in MDCK cells using standard methods.

#### Ebola viruses

Ebola virus (EBOV) *in vitro* (i.e., cell culture) experiments used biologically contained mutant Ebola-ΔVP30 based on the Zaire ebola virus (strain Mayinga, 1976), which expresses green fluorescent protein in the place of the essential Ebola VP30 protein and replicates only in cells expressing the Ebola VP30 protein^20^. Mutant viruses in the Ebola-ΔVP30 background include Ebola-ΔVP30-Δmucin^21^; and Ebola-ΔVP30-ΔssGP, which has not been described previously. Ebola-ΔVP30-Δmucin has a 151 amino acid deletion (residues 316–467) in the serine-threonine-rich mucin-like domain of the viral glycoprotein (GP). Ebola-ΔVP30-ΔssGP lacks the expression of two viral proteins, soluble GP (sGP) and small soluble GP (ssGP)^22^. Ebola GP, sGP, and ssGP are all generated from the same open reading frame: sGP, the primary product, is expressed from unedited RNA transcripts; full-length GP is expressed after transcriptional editing, which occurs at a poly-uridine tract in the genomic RNA, resulting in an additional adenosine residue in the GP transcript, a +1 frameshift, and a longer GP open reading frame (ORF); and ssGP is expressed after the addition of 2 adenosine residues in the GP ORF, resulting in a + 2 frameshift and a truncated ORF. In the Ebola-ΔVP30-ΔssGP mutant, the expression of sGP and ssGP were ablated by introducing mutations into the poly-uridine tract that produce two AGG codons in the GP ORF. The resulting transcript expresses only the full-length GP harbouring two K → R mutations and prevents the translation of sGP and ssGP. The wild-type Ebola-ΔVP30 virus and mutants thereof were rescued by reverse genetics in Vero-VP30 cells as previously described^20^ and stock virus titers were quantified using standard focus-forming unit assays. For studies involving human peripheral blood mononuclear cells (PBMC) and plasma, enrolled patients naturally acquired infection by the West African Zaire ebola virus (strain Makona).

#### WNV

West Nile virus (WNV) was isolated from a mosquito during the original outbreak in the United States in New York during 1999 (strain NY-99). WNV-NY-99 is virulent, grows to high titer in primary dendritic cells and neurons^11^, and causes lethal infection in adult wild-type C57BL/6 mice^23^. For the experiments described herein, WNV-NY-99 was rescued by reverse genetics from the two-plasmid 382 infectious clone system, as described previously^24,25^ (the reverse genetics-generated clone is referred to as ‘WNV-NY99-382’). Briefly, the 382 plasmids (pWN-AB and pWN-CG) were amplified in Stlb2 competent cells (Invitrogen) at 30 °C for 2 days on agar plates and sequences were confirmed by Sanger sequencing. To prepare genome-length WNV-NY-99 cDNA, pWN-AB was digested with NgoMIV and pWN-CG was digested with NgoMIV and XbaI (NgoMIV cleaves a natural NgoMIV site in the WNV genome, which is present in both pWN-AB and pWN-CG plasmids, and XbaI cleaves the 3’ end of the WNV genome). Then, portions of the WNV genome derived from pWN-AB and pWN-CG plasmids were ligated with T4 DNA ligase and full-length viral genomic RNA was transcribed using the AmpliScribe High Yield T7 kit (Epicentre Technologies) in the presence of a m7-GpppA RNA cap structure analog for 4 h at 37 °C. Subsequently, BHK cells were electroporated with the transcribed RNA using the Gene Pulser Xcell (BioRad), virus was harvested 3 days after electroporation; and virus stocks were generated by passaging on BHK cells, concentrated by using ultracentrifugation on sucrose, and quantified by focus forming assay, as previously described^26^. The WNV-NY99-E218A mutant virus is attenuated by virtue of an E218A amino acid substitution in NS5 gene, exhibits decreased replication in primary dendritic cells and neurons, and is avirulent in wild-type C57BL/6 J mice, although it replicates normally in Vero or BHK cells lacking the cell-intrinsic type I interferon response, including expression of IFIT family members^27,28^. For the experiments described herein, WNV-NY99-E218A was generated using a two-plasmid 382 infectious clone system (the reverse genetics-generated clone is referred to as ‘WNV-NY99-382-E218A’). To remove the potential for reversion at the E218A locus, two nucleotide substitutions were introduced into the E218 codon (*i.e*., GAG to GCA) by site-directed mutagenesis of the pWN-CG plasmid with Phusion DNA polymerase (Thermo Fisher). WNV-NY99-382-E218A was confirmed to be avirulent in wild-type C57BL/6 J mice.

#### MERS-CoV

Wild-type infectious clone of MERS-CoV (icMERS-CoV-WT), based on strain EMC-2012, and mutant viruses were rescued by reverse genetics as previously described^29^. Mutant viruses include icMERS-CoV-RFP (expressing red fluorescent protein in place of open reading frame 5, ORF5)^29^ and strains deficient in expression of non-structural protein 16 (icMERS-CoV-ΔNSP16)^30^, ORF4B (icMERS-CoV-ΔORF4B); or ORF3, ORF4A, ORF4B, and ORF5 in combination (icMERS-CoV-ΔORF3-5)^29,31^. A recombinant infectious clone derived from mouse-adapted MERS-CoV (MA1 strain)^12^ was used for *in vivo* studies. MERS-CoV MA1 differs from parental MERS-CoV EMC2012 in the 5’ UTR (∆A, nucleotide 2), nsp3 (A217V), nsp6 (T184I), nsp8 (I108L), spike (R884-RMR insertion and S885L), and ns4b (∆E45-H243, which comprises a deletion of nucleotides 26,226 to 26,821). Stock viruses were generated by passaging an aliquot of supernatant derived from reverse genetics transfection experiments on Vero81 cells, as previously described^29^, and plaque assays in Vero81 cells were used to quantitate viral stock titers using standard methods.

### Human cell lines, primary cells, and infections

*In vitro* experiments to collect samples for multi-omics analyses were performed in human cell lines or primary cells permissive to infection by influenza virus, Ebola virus, or MERS-CoV. All human cell lines and primary cells were maintained at 37 °C in an atmosphere of 5% CO_2_ in media containing antibiotics. All cell lines were regularly tested for mycoplasma. Cell line or primary cell maintenance and infections were carried out as follows.

#### Influenza virus (ICL102, ICL103, ICL104, ICL105, and ICL106)

All *in vitro* experiments with influenza viruses were performed in Calu-3 cells, a human lung bronchial epithelial cell line (kindly provided by Raymond Pickles at the University of North Carolina, Chapel Hill, NC, USA). Calu-3 cells were maintained in a 1:1 mixture of Dulbecco’s modified Eagle’s medium (DMEM) and Ham’s F12 nutrient medium (DF12) containing 10% fetal bovine serum (FBS) (DF12-FBS). Influenza infections were carried out as previously described^4,5^. Briefly, Calu-3 cells were seeded onto 6-well plates (1 × 10^6^ cells per well), provided fresh DF12-FBS after 24 hours, and infected with influenza viruses at 48 hours after plating. Prior to inoculation, Calu-3 cells were washed twice with DF12 supplemented with 0.3% bovine serum albumin (DF12-BSA) to remove residual FBS, and subsequently, monolayers were inoculated with 300 μl of DF12-BSA containing influenza viruses at a multiplicity of infection (moi) of 1 plaque forming unit (pfu) per cell (ICL102, ICL103, ICL105, and ICL106) or 3 pfu per cell (ICL104). Mock inoculations were carried out similarly with DF12-BSA lacking virus. After incubating for 45 minutes with gentle agitation every 10 minutes, inoculated (or mock-inoculated) monolayers were washed twice with 1X phosphate-buffered saline (PBS), covered with DF12-BSA containing L-(tosylamido-2-phenyl) ethyl chloromethyl ketone (TPCK)-treated trypsin (2 ml per well), and incubated until the sample collection time point.

#### Ebola virus (EHUH001, EHUH002, EHUH003, EU937001, and EHUVEC001)

Experiments were performed in Huh7 (human hepatic epithelial) or U937 (human pro-monocytic) cells stably expressing the VP30 protein (Huh7-VP30 or U937-VP30, respectively)^32,33^ or in primary human umbilical vein endothelial cells (HUVEC).

Huh7-VP30 cells were maintained in DMEM containing 10% FBS. For infection, Huh7-VP30 cells were seeded onto 6-well plates (1 × 10^6^ cells per well) and 24 hours later, monolayers were inoculated with 250 μl of DMEM-FBS containing Ebola-ΔVP30 viruses at an moi of 3 (EHUH001) or 10 (EHUH002 and EHUH003) focus-forming units (ffu) per cell, or with DMEM-FBS lacking virus for mock infections. After incubating for 1 h with gentle agitation every 15 minutes, monolayers were washed twice with 1X PBS, covered with 2 ml DMEM-FBS, and incubated until the sample collection time point.

U937-VP30 cells were maintained in RPMI 1640 medium supplemented with 10% FBS. Prior to infection, U937-VP30 cells were seeded onto 6-well plates (1 × 10^6^ cells per well) and differentiated into macrophages by treatment with phorbol 12-myristate 13-acetate (PMA, 10 ng per ml) for 24 hours, followed by an additional 24 hours without PMA treatment, as previously described^33^. Mock infections or infections with Ebola-ΔVP30 viruses (moi = 10 ffu per cell) were carried out in RPMI 1640 growth medium as just described for Huh7-VP30 cells (EU937001).

Primary HUVEC were maintained in endothelial cell growth medium (ECGM; Cell Applications, Inc.). Prior to infection, HUVEC were seeded onto 6-well plates (1 × 10^6^ cells per well) and inoculated with a replication-defective adenovirus expressing the VP30 protein (moi = 500 pfu per cell), which was produced by VectorBuilder Adenovirus VP30 packaging service. Subsequently, HUVEC cells transiently expressing the VP30 protein via the adenovirus vector were mock-inoculated or inoculated with Ebola-ΔVP30 viruses (moi = 10 ffu per cell) in ECGM as just described for Huh7-VP30 cells (EHUVEC001).

#### MERS-CoV (MCL001, MCL002, MCL003, MCL004, MCL005, MFB001, MFB002, MFB003, MHAE001, MHAE002, MHAE003, MMVE001, MMVE002, and MMVE003)

Experiments were performed in Calu-3/2B4 cells (MCL001, MCL002, MCL003, MCL004, MCL005), a clonal population of Calu-3 cells sorted for high expression of the SARS-CoV cellular receptor, angiotensin-converting enzyme 2 (kindly provided by Chien K. Tseng at the University of Texas Medical Branch, Galveston, TX)^34^; or in primary lung fibroblasts (MFB001, MFB002, MFB003), tracheobronchial epithelial cells (MHAE001, MHAE002, MHAE003), or microvascular endothelial cells (MMVE001, MMVE002, and MMVE003). Primary cells were obtained from airway specimens resected from patients undergoing surgery at the UNC-Chapel Hill and prepared by the UNC-Chapel Hill Marsico Lung Institute Tissue Procurement and Cell Culture Core. Airway specimens were collected from three individuals, each cell type (fibroblasts, tracheobronchial [airway] epithelial cells, and microvascular endothelial cells) was isolated from each specimen, and a set of the three cell types from one specimen was used in one set of experiments (*e.g*., MFB001, MHAE001, and MMVE001 experiments all used cells isolated from the same individual). Experiments were performed in cells derived from three donors.

Calu-3 clone2B4 cells were maintained in DMEM containing 20% FBS, and infections with MERS-CoV were performed as for influenza virus infections, with the following exceptions: DMEM containing 10% FBS was used for virus and mock inoculations, cells were inoculated with MERS-CoVs at an moi of 5 pfu per cell, and infected cells were incubated with DMEM containing 10% FBS until the sample collection time point.

Primary human fibroblasts were maintained in DMEM-H containing 10% FBS. Cells were seeded onto 6-well plates (1 × 10^4^ cells per well) 72 hours prior to infection, and monolayers were inoculated with 200 μl of DMEM-H with 4% FBS and MERS-CoV at an moi of 5 pfu per cell, or the same medium lacking virus for mock inoculations. After incubating for 40 minutes with gentle agitation every 10 minutes, monolayers were washed 2 times with 1X PBS, covered with 2 ml DMEM-H (4% FBS), and incubated until the sample collection time point.

Primary tracheobronchial epithelial cells were maintained in air-liquid interface (ALI) medium^35^. For infection, cells were seeded onto transwell inserts (~2.5 × 10^5^ cells per well), which were placed in transwell dishes (12 mm) and allowed to mature in ALI medium for 6 weeks. At 24 hours prior to infection, the apical surfaces were covered with 1X PBS, inserts were transferred to 12-well plates containing fresh ALI medium, and cells were allowed to equilibrate for 1 hour before removing the PBS. Immediately prior to inoculation, apical surfaces were washed with 1X PBS, and then monolayers were inoculated with 200 μl of ALI medium containing MERS-CoV at an moi of 5 pfu per cell, or with ALI medium lacking virus for mock infections. After incubating for 1.5 hours, apical surfaces were washed 2 times with 1X PBS, the final wash was removed, and cultures were incubated in 12-well plates with ALI medium until the sample collection time point.

Microvascular endothelial cells were maintained in Vasculife VEGF-MVE endothelial medium (Lifeline Cell Tech). Cells were seeded onto 6-well plates (1.15 × 10^5^ cells per well) 1 week before infection and were inoculated with MERS-CoV in a manner similar to that just described for human lung fibroblasts, except that Vasculife VEGF-MVE was used.

### Mouse primary cells and infections

*In vitro* experiments to collect samples for multi-omics analyses were performed in mouse primary cells permissive to infection by West Nile virus. All mouse primary cells were maintained at 37 °C in an atmosphere of 5% CO_2_. Primary cell maintenance and infections were carried out as follows.

#### WNV (WCN002, WCN003, WGCN002, WGCN003, WDC010, WDC011)

All experiments were performed using primary cells derived from C57BL/6 J mice.

Primary cortical neurons were generated from embryonic day 15 mouse embryos. Cortex tissues dissected from embryos were pooled together, digested with trypsin and DNase I, and dissociated by pipetting and filtering through a 70 µm filter. Then, 5 × 10^5^ cells were seeded on poly-D-lysine and laminin-coated 24-well plates in Neurobasal Plus medium with B-27 Plus supplement, and medium was changed after 2 days. After 4 days, cortical neurons were inoculated with WNV at an moi of 250 ffu/cell for 1 hour at 37 °C. Subsequently, the inoculum was removed and cells were covered with fresh medium and incubated until the sample collection time point.

Primary granule cell neurons were generated from 6-day-old mouse pups. Cerebellum tissues were dissected, pooled, digested with trypsin and DNase I, and dissociated into individual cells by triturating extensively and filtering through a 40 µm filter. Cells were seeded and inoculated with WNV as described for primary cortical neurons.

Primary bone marrow-derived dendritic cells were generated from 8-10-week-old male C57BL/6 mice using recombinant murine GM-CSF and IL-4. Briefly, bone marrow was collected and seeded in non-tissue culture (TC)-treated 100-mm dishes in RPMI medium containing 10% FBS, 1% non-essential amino acids, 1% L-glutamine, 0.02% murine GM-CSF (Peprotech, 0.1 μg/μl concentration), 0.02% murine IL-4 (Peprotech, 0.1 μg/μl concentration), and 0.1% 2-mercaptoethanol (50 mM). At days 3 and 8 after seeding, 10 ml of R10 medium was added. At day 6, 10 ml of culture supernatant was collected, centrifuged, and resuspended in 10 ml of fresh R10 DC media, and added back to the original plate. At day 10, non-adherent cells were collected, and 2 × 10^5^ cells were used for each replicate. Cells were inoculated with WNV at an moi of 500 ffu/cell for 1 hour at 37 °C. Subsequently, the inoculum was removed, and cells were covered with fresh medium and incubated until the sample collection time point.

### Treatment of cells with recombinant interferon proteins

In some experiments (IFNaHUH001, IFNaCL001, IFNaIHH001, IFNFB001, and IFNMVE001), human cells were treated with recombinant type I or type II interferon proteins (without infection) and monolayers were collected at multiple time points for transcriptomics analysis. In all cases, time-matched mock-treated controls were prepared and collected in parallel.

#### IFNaCL001

Calu-3 cells were seeded onto 6-well plates (1 × 10^6^ cells per well) and mock-treated or treated with recombinant human interferon α hybrid protein (i.e., universal type I interferon, PBL Assay Science; 1,000 U/well) in growth medium (see above). Cell monolayers were collected for transcriptomics analysis at 6, 12, and 18 h post-treatment. For each treatment and time point condition, 6 replicate samples were collected (4 of each replicate were analysed).

#### IFNaHUH001

Huh 7 cells were seeded onto 6-well plates (3 × 10^5^ cells per well) and mock-treated or treated with recombinant human interferon β (PBL Assay Science, 250 U/well) and recombinant human interferon α hybrid protein (i.e., universal type I interferon, PBL Assay Science; 250 U/well) in growth medium (see above). Cell monolayers were collected for transcriptomics analysis at 6, 12, and 18 h post-treatment. For each treatment and time point condition, 5 replicate samples were collected (4 of each replicate were analysed).

#### IFNaIHH001

IHH cells (kindly provided by Ranjit Roy, Saint Louis University, St. Louis, MO) were seeded onto 12-well plates (2 × 10^5^ cells per well) and mock-treated or treated with recombinant human interferon α hybrid protein (i.e., universal type I interferon, PBL Assay Science; 500 U/well) in DMEM supplemented with 4 mM glutamine, 10% heat-inactivated FBS and antibiotics. Cell monolayers were collected for transcriptomics analysis at 6, 12, and 18 h post-treatment. For each treatment and time point condition, 3 replicate samples were collected (all replicates were analysed).

#### IFNFB001

Primary human lung fibroblasts were seeded onto 6-well plates (1 × 10^4^ cells per well) and mock-treated or treated with recombinant human interferon α hybrid protein (i.e., universal type I interferon, PBL Assay Science; 1,000 U/well) or recombinant human interferon γ (PBL Assay Science; 1,000 U/well) in growth medium (see above). Cell monolayers were collected for transcriptomics analysis at 3, 6, and 24 h post-treatment. For each treatment and time point condition, 4–6 replicate samples were collected (all replicates were analysed).

#### IFNMVE001

Primary human lung microvascular endothelial cells were seeded onto 6-well plates (1.15 × 10^5^ cells per well) and mock-treated or treated with recombinant human interferon α hybrid protein (i.e., universal type I interferon, PBL Assay Science; 1,000 U/well) or recombinant human interferon γ (PBL Assay Science; 1,000 U/well) in growth medium (see above). Cell monolayers were collected for transcriptomics analysis at 3, 6, and 24 h post-treatment. For each treatment and time point condition, 4–6 replicate samples were collected (all replicates were analysed).

### Mouse infections and tissue collection

*In vivo* experiments to collect samples for multi-omics analyses were performed in C57BL/6 J mice (The Jackson Laboratory) infected with influenza virus or West Nile virus, or in C57BL/6 J mice expressing humanized DPP4 (C57BL/6J-hDPP4)^12^ infected with MERS-CoV.

#### Influenza virus (IM101, IM102, and IM103)

All experiments used 22-week-old C57BL/6 J mice infected with influenza virus (5 replicates per virus and time point) and time-matched mock-infected controls (3 replicates per time point). Mice were anesthetized by isofluorane inhalation and intranasally inoculated with 50 μl of PBS (mock) or PBS containing influenza viruses at a dosage of 10^4^ pfu per mouse (IM102 and IM103) or variable dosages (IM101; Note: Variable dosages were not part of the original IM101 experimental design. Rather, for all H5N1 viruses used in this experiment, back-titration revealed stock titers that were less than expected at the time the experiment was performed. Therefore, the actual dosages used to infect mice were recalculated based on the corrected stock titer values and adjusted dosages are indicated in the Experimental Design document for IM101^36^. Fresh virus stocks were generated and titered for all H5N1 viruses prior to use in IM103 or ICL103. Mouse body weights were collected daily, and mice were humanely euthanized when exhibiting severe clinical symptoms or at designated time points (1-, 2-, 4-, and 7-days post-infection) for tissue collection. Lungs were dissected and preserved for different analyses as follows: the right superior lobe was collected for virus titration and frozen at −80 °C in the absence of buffer; the right inferior lobe was collected for proteomics, metabolomics and lipidomics analyses and frozen at −80 °C in the absence of buffer; and the right middle and post-caval lobes were directly submerged in Invitrogen^TM^ RNAlater^TM^ Stabilization Solution, then placed at 4 °C overnight, followed by freezing at −80 °C.

#### West Nile virus (WCT001, WCB001, WLN002, WLN003, and WSE001)

Most experiments used 5-week-old male C57BL/6 J mice infected with West Nile virus (5 replicates per virus and time point) and time-matched mock-infected (HBSS) controls (3–5 replicates per time point), with sample collection time points of 1-, 2-, 4-, and 6-days post-infection. Experiments focused on collection of cerebral cortex (WCT001) and cerebellum (WCB001) tissues, which were obtained from the same infected animals; lymphatic tissues (popliteal lymph nodes) (WLN002 and WLN003), or serum (WSE001). All mice were humanely euthanized at the designated time points for tissue collection. For experiments with neural tissues (WCT001 and WCB001), mice were anesthetized with ketamine/xylazine and intracranially inoculated with 10 μl of HBSS (mock) or HBSS containing West Nile virus at a dosage of 100 ffu per mouse. Brains were dissected and preserved for different analyses as follows: ¼ of the cortex was collected for virus titration and frozen at −80 °C in the absence of buffer or fixed in buffered formalin for histology, while ¼ of the cortex and ½ of the cerebellum were each collected for PML and transcriptomics analyses as described above for lung tissues infected with influenza virus. For experiments with lymphatic tissues (WLN002 and WLN003), mice were anesthetized with ketamine/xylazine and inoculated subcutaneously in both hind foot pads with 20 μl of HBSS (mock) or HBSS containing West Nile virus at a dosage of 100 ffu per mouse. Draining lymph nodes were collected for PML (WLN003) or transcriptomics analyses (WLN002) in separate experiments. For HBSS-inoculated mice, popliteal lymph nodes from two mice were pooled for a total of 4 lymph nodes per time point and analysis condition. For the experiment with serum (WSE001), two groups of 6-week-old C57BL/6 J mice (3–5 replicates per time point) were administered Koolaid (20 g/L) or a cocktail of three antibiotics (1 g/L ampicillin, 1 g/L neomycin, and 0.5 g/L vancomycin) in Koolaid *ad libitum* for two weeks prior to and throughout WNV infection. For infection, mice were anesthetized with ketamine/xylazine and inoculated subcutaneously in the foot pad with 50 μl of HBSS (mock) or HBBS containing West Nile virus at a dosage of 100 ffu per mouse. Blood was collected the day before and three days after inoculation and serum was isolated and frozen at −80 °C for proteomics and metabolomics analyses.

#### MERS-CoV (MM001)

This experiment included mice infected with MERS-CoV and time-matched mock-infected controls (3–4 replicates per infection condition and time point). Fifteen- to seventeen-week-old C57BL/6J-hDPP4 mice^12^ were anesthetized with ketamine/xylazine and intranasally inoculated with 50 μl of PBS (mock) or PBS containing MERS-CoV MA1^12^ at dosages of 5 × 10^4^, 5 × 10^5^, or 5 × 10^6^ pfu per mouse. Body weights were collected daily and mice were humanely euthanized at designated time points (2-, 4-, and 7-days post-infection) for tissue collection. Lungs were dissected and preserved for different analyses as described for influenza virus.

### Blood sample collection and processing

#### Human

Blood samples were collected from human patients naturally infected with Ebola virus and processed to separate peripheral blood mononuclear cells (PBMCs) and plasma as previously described^13^. Briefly, blood samples collected in K_2_-EDTA tubes were mixed 1:1 with sterile 1X PBS, layered over 3 ml of Ficoll-Paque PLUS (GE Healthcare) in a 15-ml SepMate tube (STEMCELL Technologies), and centrifuged for 10 min at 1,200 × *g*. Following centrifugation, plasma was frozen immediately at −80 °C and PBMCs were collected and washed one time with sterile 1X PBS before lysis in TRIzol reagent (see below).

#### Mice

Blood samples were collected from mock-infected or WNV-infected mice into serum separator tubes. Serum was separated by centrifugation at 9,000 × *g* for 10 minutes. Serum aliquots were transferred to fresh tubes and stored at −80 °C until further analysis.

### Virus quantification

#### Human or mouse cells (except PBMCs)

Cell culture supernatants were collected from infected cell monolayers and frozen at −80 °C. Later, virus concentrations were quantified using standard plaque assays (influenza virus and MERS-CoV) or focus-forming unit assays (Ebola virus and WNV). Virus concentrations are reported as pfu per ml (pfu/ml) or ffu per ml (ffu/ml).

#### Mouse tissues

Mouse lung or brain tissues were collected from infected mice and stored at −80 °C in the absence of buffer. To quantify virus concentrations, tissues were thawed, weighed, and homogenized in PBS. Then, the cleared supernatants were used for standard plaque assays (influenza virus and MERS-CoV) or focus-forming unit assays (Ebola virus and WNV). Mouse serum (from WNV-infected mice) was used directly for focus-forming unit assays. Virus concentrations are reported as pfu or ffu per gram of tissue (pfu/g or ffu/g, lung or brain) or ffu per ml (ffu/ml, serum).

### Virus infectivity

#### Influenza

Infected cell monolayers, prepared as described for multi-omics sample collection experiments, were fixed with 4% paraformaldehyde for 15 minutes at room temperature. After rinsing in 1X PBS, cells were permeabilized with 0.5% Triton X-100 in 1X PBS for 5 minutes at room temperature, rinsed with 1X PBS, and blocked by incubation with 10% normal goat serum and 1% bovine serum albumin (BSA) in 1X PBS (blocking solution) for 1 hour at room temperature. Primary and secondary antibody binding were carried out in blocking solution for 1 hour at room temperature, with extensive washing with 1X PBS after each binding step. The primary antibody was rabbit polyclonal anti-H1N1 (R309; 1:1,000 dilution; this polyclonal antibody mixture recognizes epitopes of pH1N1, H5N1, and H7N9 viruses) and the secondary antibody was goat anti-rabbit conjugated with Alexa-Fluor 488 (1:250; Invitrogen). Stained cells were covered with 1X PBS. Brightfield and fluorescent images were captured with an EVOS Cell Imaging System and a 10X objective. At least three images of each type were captured across the monolayer surface at each measured time point. Percent infection was determined by counting the number of cells expressing influenza antigen and dividing by the total number of cells in all three image sets.

#### Ebola

Staining of Ebola-ΔVP30-infected cell monolayers was carried out as described for influenza, except that the primary antibody was mouse monoclonal anti-VP40 (#6) and the secondary antibody was goat anti-mouse conjugated with Alexa-Fluor 488. Percent infection was determined as described for influenza virus.

#### WNV

Percent infectivity of cell cultures was determined by flow cytometry at 24 hours after infection using an anti-WNV antibody E16^37^.

### Mouse lung haemorrhage scores

In mice infected with MERS-CoV, haemorrhage may occur in lung tissues (indicated by a change in colour from pink to dark red) as disease worsens and is an indicator of virus spread and damage. Therefore, for MERS-CoV-infected mice, gross pulmonary haemorrhage was assessed by direct observation at the time of tissue harvest. As previously described^38–41^, scores were assigned according to the appearance and extent of dark red colour using a 0–4 ordinal scale, where 0 indicates no hemorrhage in any lobe and 4 indicates extreme and complete haemorrhage in all lobes.

### Cytokine analysis

Plasma cytokines (IL6, TNF, IL10, IL1A, and IL1B) were quantified using a commercially available enzyme-linked immunosorbent assays (ELISAs; Millipore or Thermo Fisher Scientific) according to the manufacturers’ instructions. Absorbance readings were captured with a Tecan Infinite F50 plate reader, and concentrations were determined based on a standard curve generated by the Microplate Manager Software 6 version 6.0 (Bio-Rad). All samples were assessed in duplicate.

### Total RNA extraction for transcriptomics analyses

#### Human or mouse cells (except PBMCs)

At the designated time points, medium overlays were removed, and cell monolayers were washed with ice-cold PBS. Then, cells were lysed directly in the plate by addition of 1 ml cold (4 °C) TRIzol reagent (Invitrogen) and scraped off the plate surface, followed by repeated pipetting until the lysate exhibited homogenous colour and consistency. Homogenized lysates were transferred to a fresh 2 ml tube, vortexed thoroughly, incubated for 5 minutes at room temperature, snap-frozen on dry ice, and transferred to −80 °C. RNA was isolated from TRIzol lysates by the commercial vendor that carried out transcriptomics analyses (ArrayStar).

#### Mouse tissues

Frozen tissues suspended in RNAlater^TM^ Stabilization Solution (stored at −80 °C) were thawed, weighed, and transferred to a 2 ml tube containing 1 ml of TRIzol (Invitrogen) and a 5 mm metal homogenization bead. Tissues were homogenized using a Qiagen TissueLyser II (30-Hz oscillation frequency for 3 min). Tissues homogenized in TRIzol were incubated for 10 minutes at room temperature and then centrifuged at 4 °C for 10 minutes at 12,000 × *g*. The clarified supernatants were transferred to a fresh tube, snap-frozen on dry ice, and transferred to −80 °C. RNA was isolated from TRIzol homogenates by the commercial vendors that carried out transcriptomics analyses (ArrayStar or Ambry Genetics).

#### Human PBMCs

PBMC pellets were suspended in 1 ml of TRIzol reagent, incubated for 10 minutes at room temperature, and frozen at −80 °C. To extract RNA, thawed TRIzol lysates were homogenized with QiaShredder columns (Qiagen),mixed with 200 μl of chloroform, vortexed, and incubated for 10 minutes on ice. Phase separation was carried out by centrifugation (12,000 × *g* for 15 min at 4 °C), and total RNA was extracted from the upper phase with miRNEasy columns (Qiagen) according to the manufacturer’s instructions.

### Microarray analysis

For all experiments carried out in cell lines, primary human or mouse cells, or mouse tissues, transcriptomics analyses were performed by commercial vendors providing microarray analysis services (ArrayStar Inc or Ambry Genetics). Frozen TRIzol cell lysates or tissue homogenates (prepared as just described) were shipped to the vendor, where total RNA extraction and quality control, and microarray analyses and quality control were performed.

The following microarray platforms were used:

- Agilent-026652 Whole Human Genome Microarray 4x44K v2 was used for mRNA transcriptomics analyses of all human cell line or primary cell experiments.
- Agilent-046064 Unrestricted Human miRNA Microarray v19.0 was used for microRNA transcriptomics analyses of all human cell line or primary cell experiments.
- Agilent-026655 Whole Mouse Genome Microarray 4x44K v2 was used for mRNA transcriptomics analyses of all mouse primary cell or tissue experiments.
- Agilent-028005 SurePrint G3 Mouse GE 8x60K Microarray was used for mRNA transcriptomics analysis of mouse tissues in one experiment only (IM103).
- Agilent-046065 Mouse miRNA Microarray v19.0 was used for microRNA transcriptomics analyses of all mouse primary cell or tissue experiments.

RNA was hybridized to the appropriate arrays and scanned on an Agilent DNA microarray scanner using the XDR setting. Raw images were processed using the Agilent Feature Extraction software by the vendor (ArrayStar Inc or Ambry Genetics).

### Viral genomic RNA quantification

Ebola virus genomic RNA was quantified in total PBMC RNA extracts (isolated from human blood and extracted as described above) using the Ebola 2014 outbreak genesig qRT-PCR kit (which detects the nucleoprotein gene of the Ebola virus Makona strain) and oasig one-step qRT-PCR master mix (Primerdesign), according to the manufacturer’s instructions^13^.

### RNA-Seq analysis

Total RNA from human PBMCs, prepared as described above, were shipped to ISMMS for RNA-Seq analyses.

Transcriptomics analysis of human PBMCs derived from patients naturally infected with Ebola virus and healthy volunteers was carried out using RNA-Seq as previously described^13^. Total RNA (extracted as described above) was treated with 1 U of Baseline Zero DNase (Epicentre) at 37 °C for 30 min, cleaned with AMPureXP beads (Beckman-Coulter), and eluted in nuclease-free water. RNA quality was assessed with an Agilent Bioanalyzer and RNA quantity was determined using the Qubit RNA Broad Range Assay kit (Thermo Fisher). Library preparation was carried out with up to 500 ng of each DNase-treated sample as follows: (*i*) Globin and ribosomal RNAs were depleted with Globin-Zero Gold rRNA Removal Kit (Illumina), the remaining RNA was purified with AMPureXP beads, and ribosomal RNA depletion was confirmed using an Agilent Bioanalyzer; and (*ii*) RNA was fragmented and libraries were prepared using the TruSeq Stranded Total RNA Library Prep Kit (Illumina) according to the manufacturer’s instructions. The resultant libraries, with barcoded adaptors for each sample, were pooled and sequenced on the Illumina HiSeq. 4000 platform in a 100-bp paired-end read run format. Read sequences were demultiplexed and trimmed at the 3’ end either after reaching a base with a PHRED quality score lower than 10, or after encountering 15 bases with a PHRED score lower than 28. Cutadapt v1.9.1^42^ was used to remove adaptor sequences and reads less than 50 nt (for paired-end 100 nt) in length were eliminated from further analysis. Full-length adapter-trimmed reads were mapped to the human (hg38) and viral (EBOV/G3683/KM034562.1) reference genomes using STAR v2.5.1b^43^ with the corresponding gene annotations (Gencode GRCh37/V23 for the human genome). Total mapped read counts per gene were determined using featureCounts v1.5.0-p1^44^ with default settings.

### Protein, metabolite, and lipid extraction for proteomics, metabolomics, and lipidomics analyses

#### Human or mouse cells (except PBMCs)

Proteins, metabolites, and lipids were extracted simultaneously using an established chloroform/methanol extraction procedure (MPLEx)^45–47^. Briefly, at the indicated time points, medium was removed and monolayers were washed with a rapid quenching solution (60% methanol [v/v] and 0.85% ammonium bicarbonate in dH2O [w/v], stored at −80 °C until just before use). Cells were scraped into 150 μl of ice-cold 150 mM ammonium bicarbonate and transferred to 2 ml siliconized SafeSeal microcentrifuge tubes (Sorenson Bioscience, Inc.). Then, 600 µl of a 2:1 chloroform/methanol solution (stored at −80 °C until just before use) was added and samples were vortexed vigorously for 10 seconds and centrifuged at 9,000 × *g* for 10 minutes at 4 °C. The upper (aqueous/methanol) and lower (organic/chloroform) phases (metabolites and lipids, respectively) were transferred to clean siliconized SafeSeal microcentrifuge tubes and evaporated to dryness in a speedvac. The protein interlayer was transferred to a clean siliconized SafeSeal microcentrifuge tube, washed with 200 μl of ice-cold 100% methanol, and air-dried. Protein, metabolite, and lipid extracts were stored at −80 °C until further analysis.

#### Mouse lung, brain, or lymph node

Frozen tissues were thawed, homogenized in 300 µl of ice-cold 150 mM ammonium bicarbonate, and centrifuged at 15,000 × g for 10 minutes. Supernatants were transferred to siliconized SafeSeal microcentrifuge tubes, mixed with 600 µl of a 2:1 chloroform/methanol solution (stored at −80 °C until just before use) by vortex for 10 seconds, and incubated for 10 minutes at room temperature. Samples were centrifuged at 9,000 × *g* for 10 minutes and metabolite (upper/aqueous), lipid (lower/organic), and protein (interlayer) extracts were collected as described for human or mouse cells (see above).

#### Human plasma

Lipids and metabolites were extracted simultaneously using the standard MPLEx protocol procedure^13,45–47^. Briefly, 150 μl of plasma (thawed from −80 °C) were mixed with 600 μl of a 2:1 chloroform/methanol solution (stored at −20 °C until just before use) in siliconized SafeSeal microcentrifuge tubes, vortexed vigorously, incubated at room temperature for 20 minutes, and centrifuged at 12,000 × *g* for 10 min. Aqueous (metabolite) and organic (lipid) phases were transferred to fresh siliconized tubes, and evaporated to dryness in a speedvac. Protein interlayers were discarded. To obtain proteins, plasma (20 μl per sample) was depleted of the 14 most abundant plasma proteins using Seppro IgY14 spin columns (Sigma-Aldrich) according to the manufacturer’s instructions^13^. Immunodepleted eluates were transferred to Amicon Ultra 4 Centrifugal Filter Units (Millipore) and centrifuged at 3,260 ×*g* until eluates were concentrated to approximately 150 μl. Concentrated eluates were mixed with urea to a final concentration of 8 M and incubated at room temperature for 15 min. Dried metabolite and lipid extracts and protein extracts in 8 M urea were stored at −80 °C until further analysis.

#### Mouse plasma

Metabolites were extracted using the MPLEx protocol procedure, as described for human plasma (see above). The protein interlayer generated by the MPLEx extraction procedure was collected as described for other mouse tissues (see above).

### Proteomics analysis

Protein extracts, prepared as described above, were shipped to PNNL for proteomics analyses.

#### Sample preparation

Sample preparation involved the initial blocking and randomization of protein extracts. Tubes were opened to facilitate the evaporation of any remaining solvent from the MPLEx extraction process. Subsequently, the samples were introduced into an epMotion automated liquid handler (Eppendorf), where 200 µl of 8 M urea in 100 mM ammonium bicarbonate (pH 8) was dispensed into each tube. The tubes were then taken out, subjected to vortex and bath-sonication to ensure proper solubilization, lightly centrifuged to settle the liquid, and repositioned in the epMotion. To determine the protein concentration of each sample, a bicinchoninic acid (BCA) assay (Thermo Scientific) was conducted. Spreadsheets were utilized for calculating total protein and determining the trypsin mass required for digestion. Dithiothreitol was introduced to each sample at a final concentration of 10 mM, and the samples underwent denaturation and reduction through an incubation period of 1 hour at 37 °C with constant shaking at 800 rpm in a Thermomixer R (Eppendorf). Following this, iodoacetamide was added at a final concentration of 20 mM, and sample alkylation was carried out for 1 hour at room temperature with constant shaking at 800 rpm in a dark environment. The samples were then diluted 8-fold with 50 mM ammonium bicarbonate and 1 mM calcium chloride. Sequence-grade trypsin (Promega) was added at a 1:50 enzyme-to-protein ratio, and the samples were incubated for 3 hours at 37 °C with constant shaking at 450 rpm in a Thermomixer with a ThermoTop. Subsequent to digestion, desalting of the samples was performed using a 4-probe positive pressure Gilson GX-274 ASPEC™ system (Gilson Inc.) with Discovery C18 50 mg/1 ml solid-phase extraction tubes (Supelco). The desalting protocol involved the addition of 3 ml of methanol for conditioning, followed by 2 ml of 0.1% trifluoroacetic acid (TFA) in water. The samples were acidified, loaded onto each column, and washed with 4 ml of 95:5 water:acetonitrile, 0.1% TFA. Clean peptides were eluted using 1 ml of 80:20 acetonitrile:water, 0.1% TFA. Subsequently, the sample volumes were concentrated to approximately 100 µl using a Speed Vac, and a final BCA assay was conducted to determine the peptide concentration. The samples were then diluted to a concentration of 0.25 µg/µl for mass spectrometry (MS) analysis.

#### High pH RP C-18 fractionation of peptide samples

For certain experiments, the creation of unique peptide accurate mass and time (AMT) tag databases was undertaken. Before conducting LC-MS/MS analysis for both peptide identification and AMT tag database establishment, samples from these experiments underwent offline high pH reversed-phase fractionation, as detailed previously^48^. To outline the process briefly, mock-infected and virus-infected samples were individually pooled and adjusted to a volume of 900 µl using 10 mm ammonium formate buffer (pH 10.0). These samples were then resolved on a XBridge C18 column (250 × 4.6 mm, 5 μM) with a 4.6 × 20 mm guard column of the same material (Waters). The separation was carried out at a flow rate of 0.5 ml/min utilizing an Agilent 1100 series HPLC system (Agilent Technologies). The mobile phases employed were (A) 10 mM ammonium formate, pH 10.0, and (B) 10 mM ammonium formate, pH 10.0/acetonitrile (10:90). The gradient transitioned from 100% A to 95% A within the initial 10 minutes, followed by shifts from 95% A to 65% A between minutes 10 and 70, 65% A to 30% A during minutes 70 to 85, maintenance at 30% A from minutes 85 to 95, re-equilibration with 100% A from minutes 95 to 105, and a consistent 100% A composition from minute 105 onward. Fractions were collected every 1.25 minutes, resulting in 96 fractions collected over the entire gradient. All fractions underwent partial drying under vacuum, and after 40 minutes (to ensure exclusion of any contamination peaks), every 5th fraction was combined. The combined fractions were then completely dried, and 15 µl of 25 mM ammonium bicarbonate was added to each fraction for storage at −20 °C until LC-MS/MS analysis.

#### Capillary LC-MS analysis of peptide samples

Peptide samples were analysed using a variety of liquid chromatography (LC) methods depending on the nature of the experiment. The different LC methods are summarized below, and the specific LC method used for each proteomics experiment is given in Supplementary Table 3.

- Waters nano-Acquity M-Class: Ultra-performance liquid chromatograph (UPLC) with a dual pumping configuration specifically designed for on-line trapping of a 5-μl injection at 3 μl/min, featuring reverse-direction elution onto the analytical column at 300 nl/min. The columns used were packed in-house using 360 μm outer diameter fused silica (Polymicro Technologies Inc.) with 1-cm sol-gel frits for media retention^49^ These columns contained Jupiter C18 media (Phenomenex) with a particle size of 5 μm for the trapping column (100 μm inner diameter × 4 cm long) and 3 μm for the analytical column (75 μm i.d. × 70 cm long). The mobile phases utilized were (i) 0.1% formic acid in water and (ii) 0.1% formic acid in acetonitrile. The gradient profile for elution followed a specified pattern. (min, %ii): 0, 1; 2, 8; 20, 12; 75, 30; 97, 45; 100, 95; 110, 95; 115, 1; 150, 1.
- Agilent custom-built: A high-performance liquid chromatograph (HPLC) system was employed, featuring a custom configuration incorporating 100-ml Isco Model 100DM syringe pumps (Isco, Inc.), 2-position Valco valves (Valco Instruments Co.), and a PAL autosampler (Leap Technologies). This setup enabled fully automated sample analysis across four distinct HPLC columns^50^. In-house manufacturing was undertaken for reversed-phase capillary HPLC columns. These columns were created by slurry packing 3-µm Jupiter C18 stationary phase (Phenomenex) into a 60-cm length of 360 µm o.d. × 75 µm i.d. fused silica capillary tubing (Polymicro Technologies Inc.). A 1-cm sol-gel frit (an unpublished PNNL variation of that described in Maiolica *et al*.^49^) for retention of the packing material. The mobile phase comprised 0.1% formic acid in water (A) and 0.1% formic acid in acetonitrile (B). Degassing of the mobile phase was achieved using an in-line Degassex Model DG4400 vacuum degasser (Phenomenex). The HPLC system underwent equilibration at 10 kpsi with 100% mobile phase A. Subsequently, a mobile phase selection valve was switched 50 minutes after injection, creating a near-exponential gradient as mobile phase B displaced A in a 2.5 ml active mixer. To control the gradient speed under constant pressure operation (10 kpsi), approximately 20 µl/min of flow was split using a 30-cm length of 360 µm o.d. × 15 µm i.d. fused silica tubing before reaching the injection valve (5 µl sample loop). The split flow effectively managed the gradient speed, and the flow through the capillary HPLC column, when equilibrated to 100% mobile phase A, was approximately 500 nl/min.

- ISCO custom-built: This custom-built LC system combines two Agilent 1200 nanoflow pumps, one Agilent 1200 cap pump (Agilent Technologies), various Valco valves (Valco Instruments Co.), and a PAL autosampler (Leap Technologies). The incorporation of custom software enabled full automation, facilitating parallel event coordination, and achieving near 100% MS duty cycle using two trapping and analytical columns. In-house preparation involved the creation of reversed-phase columns by slurry packing 3 µm Jupiter C18 (Phenomenex) into 40 cm × 360 µm o.d. × 75 µm i.d. fused silica (Polymicro Technologies Inc.) and a 1-cm sol-gel frit was employed for media retention during the packing process^51^. For the preparation of trapping columns, a similar approach was employed, involving the slurry packing of 5-µm Jupiter C18 into a 4-cm length of 150 µm i.d. fused silica, fritted on both ends. The mobile phases utilized were 0.1% formic acid in water (A) and 0.1% formic acid in acetonitrile (B), operating at a flow rate of 300 nl/min. The gradient profile for mobile phase B was programmed as follows (min, %B): 0, 5; 2, 8; 20, 12; 75, 35; 97, 60; 100, 85. Sample injections (5 µl) underwent trapping and washing on the trapping columns at 3 µl/min for 20 minutes before alignment with the analytical columns. Data acquisition was intentionally delayed by 15 minutes relative to the gradient start and end times to accommodate the column dead volume, ensuring the tightest possible overlap in two-column operation. The use of two-column operation also provided the flexibility for columns to be ‘washed’ (shortened gradients) and re-generated off-line, without incurring any cost to the duty cycle.
  Similarly, the LC systems were coupled to various MS instrumentation with specific methods depending on the nature of the experiment. The different MS instrumentations used are summarized below, and the specific MS instrument used for each proteomics experiment is given in Supplementary Table 3.
- An LTQ Orbitrap mass spectrometer (ThermoScientific) utilized a customized ion electrospray ionization (ESI) interface. For electrospray emitters, custom-made versions were crafted using 150 µm o.d. × 20 µm i.d. chemically etched fused silica^52^. The heated capillary temperature and spray voltage were 200 °C and 2.2 kV, respectively.
- A Velos Orbitrap mass spectrometer (ThermoScientific) was outfitted with a custom ion electrospray ionization (ESI) interface. Electrospray emitters were custom-made as described was equipped with a personalized ion electrospray ionization (ESI) interface. Similar to the LTQ Orbitrap, electrospray emitters were custom-made following the previously described process^52^. However, for this instrument, the heated capillary temperature and spray voltage were set at 350 °C and 2.2 kV, respectively.

For both the LTQ Orbitrap and Velos Orbitrap mass spectrometers, the data acquisition spanned 100 minutes, commencing 65 minutes after the injection of the sample (15 minutes into the gradient). Orbitrap spectra (with an AGC set at 1 × 10^6) were captured within the 400–2000 m/z range at a resolution of 60k. Subsequently, data-dependent ion trap MS/MS spectra (with AGC set at 1 × 10^4) were acquired for the 10 most abundant ions, employing a 2 m/z isolation width and 35% collision energy. To avoid redundancy, a dynamic exclusion time of 45 seconds was implemented, discriminating against previously analyzed ions falling within the range of −0.55 and 1.55 atomic mass units.

#### Data processing

Proteomics data were processed by one of two data analysis pipelines. For construction of AMT tag libraries, LC-MS/MS data from fractionated samples were processed to identify peptides based on their MS/MS spectra. For quantitative LC-MS analyses, the data were processed according to the AMT tag approach.

- For the construction of the AMT tag database, LC–MS/MS raw data underwent conversion into dta files using Bioworks Cluster 3.2 (Thermo Fisher Scientific). The MSGF + algorithm was then employed to search MS/MS spectra against the Human Uniprot database dated 2016-04-13, containing 20,154 entries, in addition to the Zaire_Ebola virus sequence from 2014-07-10, which included 7 viral protein entries. The key search parameters included a ± 20 ppm tolerance for precursor ion masses, +2.5 Da and −1.5 Da window on fragment ion mass tolerances, utilization of MSGF + high resolution HCD scoring model, no limit on missed cleavages but a maximum peptide length of 50 residues, partial or fully tryptic search, variable oxidation of methionine (15.9949 Da), and fixed alkylation of cysteine (carbamidomethyl, 57.0215 Da). To maintain accuracy, a decoy database searching methodology was implemented to control the false discovery rate (FDR) at the unique peptide level, ensuring it remained below 1%. Subsequently, the FDR at the protein level was regulated to be less than 0.5%, calculated as (% FDR = ((reverse identifications∗2)/total identifications)∗100).
- The identification and quantification of detected peptide peaks followed the label-free AMT tag approach^13,53^. Internally developed informatics tools, featuring algorithms for peak-picking and determining isotopic distributions and charge states, were employed to process the LC-MS data. This processing involved correlating the resultant LC-MS features with an AMT tag database. Downstream, all potentially identified peptides were visualized using VIPER, an automated program that facilitated the correlation of LC-MS features with the peptide identifications housed in the AMT tag database database^54^.

### Metabolomics analysis

Metabolite extracts, prepared according to the previously outlined procedure, were shipped to PNNL for metabolomics analyses. The dried extracts underwent chemical derivatization using a modified version of the protocol employed for creating FiehnLib^55^. In brief, the extracts were dried once more to eliminate any residual moisture. To safeguard carbonyl groups and reduce the number of tautomeric isomers, 20 μl of methoxyamine in pyridine (30 mg/ml) were added to each sample, followed by vortexing for 30 s and an incubation period at 37 °C with shaking (1,000 rpm) lasting 90 minutes. Subsequently, the sample vials were inverted once to capture any solvent condensation at the cap surface, followed by a brief centrifugation at 1,000 × g for 1 minute. For the derivatization of hydroxyl and amine groups into trimethylsilyated (TMS) forms, 80 μl of N-methyl-N-(trimethylsilyl) trifluoroacetamide (MSTFA) with 1% trimethylchlorosilane (TMCS) were added to each vial. This was followed by vortexing for 10 s and an incubation period at 37 °C with shaking (1,000 rpm) for 30 minutes. Again, the sample vials were inverted once, followed by centrifugation at 1,000 × g for 5 minutes. Analysis was conducted using an Agilent GC 7890 A coupled with a single quadrupole MSD 5975 C (Agilent Technologies). An HP-5MS column (30 m × 0.25 mm × 0.25 μm; Agilent Technologies) was employed for untargeted metabolomics analyses. The sample injection mode was splitless, with 1 μl of each sample injected. The injection port temperature was held at 250 °C throughout the analysis. The GC oven was initially set at 60 °C for 1 minute after injection, followed by a temperature increase to 325 °C by 10 °C/min, with a subsequent 10-minute hold at 325 °C. The helium gas flow rate was determined by the Agilent Retention Time Locking function, and data were collected over the mass range 50–550 m/z. A mixture of FAMEs (C8-C28) was analyzed once daily with the samples for retention index alignment during subsequent data analysis.

GC-MS raw data files were processed using the Metabolite Detector software, version 2.5.2 beta^56^. The Agilent D files were first converted to netCDF format using Agilent Chemstation and then to binary files using Metabolite Detector. Retention indices of detected metabolites were calculated based on the analysis of the FAMEs mixture, followed by chromatographic alignment across all analyses after deconvolution. Metabolites were initially identified by matching experimental spectra to a PNNL-augmented version of FiehnLib, which contains spectra and validated retention indices for over 1,000 metabolites, using a Metabolite Detector match probability threshold of 0.6 (combined retention index and spectral probability). Manual validation of metabolite identifications was performed to minimize deconvolution errors during automated data processing and eliminate false identifications.

The NIST 17 GC-MS library^51^ was employed for cross-validating the spectral matching scores obtained using the Agilent library and to provide identifications for unmatched metabolites. The three most abundant fragment ions in the spectra of each identified metabolite were automatically determined by Metabolite Detector, and their summed abundances were integrated across the GC elution profile. Fragment ions resulting from trimethylsilylation (i.e., m/z 73 and 147) were excluded from the determination of metabolite abundance. A matrix encompassing identified metabolites, unidentified metabolite features (characterized by mass spectra and retention indices and assigned as ‘unknown’), and their abundances was generated for statistical analysis. Features originating from GC column bleeding were eliminated from the data matrices before further processing and analysis.

The data were imported into MatLab R2014a and subjected to log_2_ transformation. Outliers were assessed using Pearson correlation and robust Mahalanobis distance, and the log_2_ values were subsequently median-centered.

### Lipidomics analysis

Lipid extracts, prepared as detailed earlier, were sent to PNNL for lipidomics analyses. The dried lipid extracts underwent analysis by LC-MS/MS using a Waters NanoAcquity UPLC system interfaced with a Velos Orbitrap mass spectrometer (Thermo Scientific). The electrospray ionization emitter and MS inlet capillary potentials were set at 2.2 kV and 12 V, respectively.

After reconstituting lipid extracts in 200 μl of methanol, 7 μl of each sample was injected and separated over a 90-minute gradient elution. The mobile phases consisted of acetonitrile/H_2_O (40:60) containing 10 mM ammonium acetate (mobile phase A) and acetonitrile/isopropanol (10:90) containing 10 mM ammonium acetate (mobile phase B), operating at a flow rate of 30 μl/min. Analysis was conducted in both positive and negative ionization modes, with a full scan range of 200–2,000 m/z. Higher-energy collision dissociation (HCD) and collision-induced dissociation (CID) were applied to the top 6 most abundant ions to ensure comprehensive coverage of the lipidome. A normalized collision energy of 30 and 35 arbitrary units for HCD and CID, respectively, was employed. Both CID and HCD were set with a maximum charge state of 2 and an isolation width of 2 m/z units. A Q value of 0.18 was utilized for CID activation.

Confident lipid identifications were accomplished through the utilization of LIQUID^57^, a tool that facilitates the examination of tandem mass spectra for diagnostic ion fragments along with associated hydrocarbon chain fragment information. The assessment included a thorough examination of the isotopic profile, extracted ion chromatogram, and mass measurement error of precursor ions for each lipid species.

To enhance the quantification of lipids, a reference database was established, comprising lipid names, observed m/z values, and retention times for lipids identified from the MS/MS data. Lipid features obtained from each analysis were aligned with the reference database based on their m/z and retention time using MZmine 2^58^. The alignment process was followed by manual verification of aligned features, and peak apex intensity values were extracted for subsequent statistical analysis.

Both positive and negative ionization data were subjected to separate analyses at all stages. The normalization and outlier detection procedures employed were consistent with those described for proteomics.

### Sample extraction for ChIP-Seq

ChIP-Seq samples were extracted from Calu-3 cells infected with influenza or coronaviruses using the EpiTect ChIP OneDay Kit (Qiagen) as previously described^59^. Briefly, at the designated time points, formaldehyde was added directly to medium covering monolayers to a final concentration of 1%, and cells were incubated at 37 °C for 10 minutes. Following incubation, cells were washed twice with ice-cold PBS, scraped into PBS, and centrifuged at 400 × *g* for 5 minutes. The cell pellet was resuspended in 200 μl of SDS lysis buffer (1% SDS, 10 mM EDTA, and 50 mM Tris, pH 8.1; Millipore), incubated at 4 °C for 10 minutes, and frozen at −80 °C. Later, thawed cells were lysed and sonicated to generate chromatin fragments of 250–1,000 base pairs in length. Sonicated samples were immunoprecipitated with anti-H3K4me3 (Qiagen) and anti-H3K27me3 (Qiagen) antibodies.

### ChIP-Seq analysis

ChIP-Seq analysis was performed as previously described^59^. Briefly, libraries were prepared from immunoprecipitated DNA (see above) by using the TruSeq ChIP Library Preparation Kit (Illumina) and sequenced on an Illumina HiSeq instrument. Sequencing data were analysed with the CLC Genomics Workbench (Qiagen) with the Histone ChIP-Seq plugin. Paired end reads were mapped against the human GRCh37/hg19 reference genome using a stringent alignment setting (mismatch cost = 2), and peaks were called against time-matched mock reference reads.

### Sample extraction for MeDIP-Seq

MeDIP-Seq samples were extracted from Calu-3 cells infected with influenza or coronaviruses using the PureLink Genomic DNA Mini Kit (Invitrogen) as previously described^59^. Briefly, at the designated time points, cells were washed with PBS, trypsinized to create a single cell suspension, and pelleted by centrifugation (250 × *g*, 5 minutes). Cells were resuspended in 200 μl of growth medium (to inactivate trypsin), DNA was extracted according to the manufacturer’s instructions, and the eluted DNA was frozen at −80 °C.

### MeDIP-Seq analysis

MeDIP-Seq analysis was performed as previously described^59^. Briefly, libraries were prepared from immunoprecipitated DNA (see above) using the TruSeq DNA Methylation Kit (Illumina) and sequenced on an Illumina HiSeq instrument. Sequencing data were analysed with the CLC Genomics Workbench (Qiagen) with the Bisulfite Sequencing plugin. Paired end reads were mapped against the human GRCh37/hg19 reference genome using a stringent alignment setting (mismatch cost = 2), and methylation levels were called against time-matched mock reference reads.

### Quantification and statistical analysis

All transcriptomics, proteomics, metabolomics, and lipidomics datasets were statistically analysed at PNNL. ChIP-Seq and MeDIP-Seq datasets were analysed at UNC-Chapel Hill.

#### Microarray data

Extracted raw data (generated by the vendor, *i.e*., ArrayStar Inc or Ambry Genetics) were background corrected using the maximum likelihood estimation for normal-exponential convolution model^60^ with an offset of 50, as implemented in Bioconductor’s^61^ *limma* package^62^. Probes were required to pass Agilent QC flags for all replicates of at least one infected time point. Data were then subjected to quantile normalization using the ‘normalizeBetweenArrays’ method available in *limma* package^62^, which includes log2 transformation of the data. Replicate probes were mean summarized into a single RNA measure. To identify differentially expressed gene products, we again used the *limma* package to calculate a p-value based on a moderated t-statistic and adjusted to correct for multiple hypothesis testing using the method of Benjamini and Hochberg^63^ that controls the False Discovery Rate (FDR). A gene product in an infected condition was considered differentially expressed (when compared to its time-matched mock-infected control) if its q-value (*i.e*., the FDR-adjusted p-value) was ≤0.05 and the absolute value of its fold-change was ≥1.5 (non-log transformed).

#### RNA-Seq data

RNA-Seq data were obtained from PBMCs of human patients naturally infected with Ebola virus and healthy volunteers were analysed as described previously^13^ using the Bioconductor *limma* package^61^. Briefly, raw fragment (*i.e*., paired-end read) counts were filtered to remove low-expressed genes as follows: (*i*) The RSEM package (with default settings in strand-specific mode) was used to convert gene counts to fragments per kb per million reads (FPKM), and only genes with >1 FPKM in >50% of samples were retained for further analysis; and (*ii*) Genes with <200 nucleotides in length or fewer than 50 total reads in all samples were removed. After removing low-expressed genes, raw fragment counts from remaining genes were combined into a numeric matrix and normalization was performed with the weighted trimmed mean of M-values (TMM) method and voom mean-variance transformation. Pairwise comparisons were made from data fitted to a design matrix comprising all sample groups. To control the False Discovery Rate, eBayes adjusted *p*-values were corrected using the Benjamini-Hochberg method^63^. Comparisons with q < 0.01 were considered significantly different.

#### Proteomics data

The peak intensity values (i.e., abundances) for the final peptide identifications were processed in a series of steps using MatLab® R2013b including quality control, normalization, protein quantification, and comparative statistical analyses. Peptide abundances were transformed to the log10 scale. Missing data values were not imputed. Quality control processing was performed to identify and remove peptides with an insufficient amount of data across the set of samples using the IMD-ANOVA algorithm^64^. Outlier detection was performed with the rMd-PAV algorithm to identify and remove LC-MS runs showing significant deviation from the standard behaviour of all LC-MS analyses based on distributional properties of the data^65^. LC-MS runs were identified as outliers at a significance level of 0.0001. Peptides were normalized using a statistical procedure for the analysis of proteomic normalization strategies (SPANS) that identified a rank invariant peptide selection approach and data scaling factor, which introduces the least amount of bias into the dataset^65^. Peptides were evaluated for quantitative changes using analysis of variance (ANOVA) with a Dunnett multiple testing correction; and for qualitative changes using a G-test with a Bonferroni multiple test correction^66^. Proteins were quantified using Bayesian selection across peptide signatures to identify the dominant statistically significant patterns^67^, and then peptides selected for protein quantification were averaged to a protein level estimate using a reference-based roll-up method^68,69^. Differentially expressed proteins were identified by comparing the appropriate time-matched mock with infected samples using the same quantitative and qualitative approaches summarized previously for peptide-level data using a threshold of p ≤ 0.05.

#### Metabolomics data

The peak intensity values (i.e., abundances) for the final metabolite identifications were processed in a series of steps using MatLab® R2013b including quality control, normalization and comparative statistical analyses. Metabolite abundances were transformed to the log_10_ scale. Missing data values were not imputed. Quality control processing was performed to identify and remove metabolites with an insufficient amount of data across the set of samples using the IMD-ANOVA algorithm^64^. Outlier detection was performed with the rMd-PAV algorithm to identify and remove GC-MS runs showing significant deviation from the standard behavior of all GC-MS analyses based on distributional properties of the data^65^. GC-MS runs were identified as an outlier at a significance level of 0.0001. Metabolites were normalized across biological replicates using median centering. Comparative statistical analyses of time-matched mock with infected samples was done using a Dunnett-adjusted t-test to assess differences in metabolite average abundance and a G-test to assess associations among factors due to the presence/absence of response^66^.

#### Lipidomics data

The peak intensity values (i.e., abundances) for the final lipid identifications were processed in a series of steps using MatLab® R2013b including quality control, normalization, and comparative statistical analyses. Lipid abundances were transformed to the log_10_ scale. Missing data values were not imputed. Quality control processing was performed to identify and remove lipids with an insufficient amount of data across the set of samples using the IMD-ANOVA algorithm^64^. Outlier detection was performed with the rMd-PAV algorithm to identify and remove LC-MS runs showing significant deviation from the standard behaviour of all LC-MS analyses based on distributional properties of the data^65^. LC-MS runs were identified as an outlier at a significance level of 0.0001. Lipids were normalized across biological replicates using median centering. Comparative statistical analyses of time-matched mock with infected samples was done using a Dunnett-adjusted t-test to assess differences in lipid average abundance and a G-test to assess associations among factors due to the presence/absence of response^66^.

#### ChIP-Seq data

Sequencing data were analysed with the CLC Genomics Workbench (Qiagen) with the Histone ChIP-Seq plugin as previously described^59^. To identify specific genomic regions where histone modifications were enriched, regions with significant fit with the peak shape were called at *p* < 0.05.

#### MeDIP-Seq data

Sequencing data were analysed with the CLC Genomics Workbench (Qiagen) with the Bisulfite Sequencing plugin as previously described^59^. Differential methylation was compared using a Fisher exact test (*p* < 0.05).

## Data Records

**Fig. 4:**
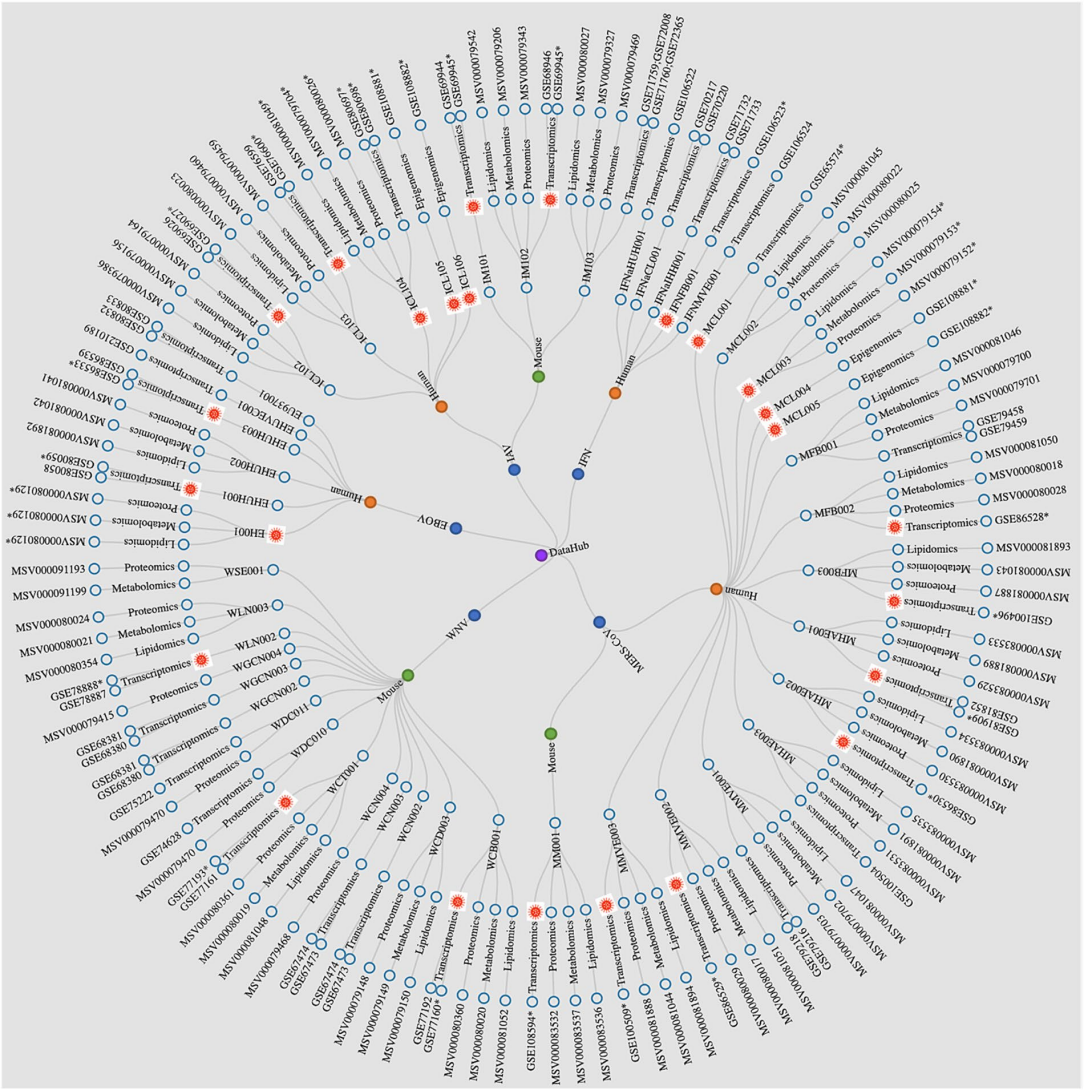
Radial representation of all project raw measurement database submission accessions, located at GEO and/or MassIVE domain repository, reporting >20,000 publicly accessible NIH supported dataset files is shown. The red icons represent a primary dataset that has been previously reported in a corresponding journal article publication. Of the 136 raw measurement dataset submissions, from the 45 experimental processed data collections, only 34 (25%) have been reported in a previous publication.

For all experiments under the OMICS-LHV project, data records are comprised of primary experimental metadata (describing the experimental design, data outputs, and phenotypic data associated with each experiment), links to raw multi-omics dataset accessions that have been deposited into public repositories (NCBI GEO^70,71^ or MassIVE^72^), and statistically processed multi-omics dataset files. Further description of each data record component is provided below. Data deposited into public repositories were submitted based on the repository’s requirements and included sufficient information to allow users to find the datasets, understand the experiment that was performed, and determine the potential for individual reuse. Raw multi-omics datasets can be accessed at the appropriate public repository (accession numbers provided below), and all public repository accessions associated with all datasets reported herein are shown in Fig. 4.

**Fig. 5:**
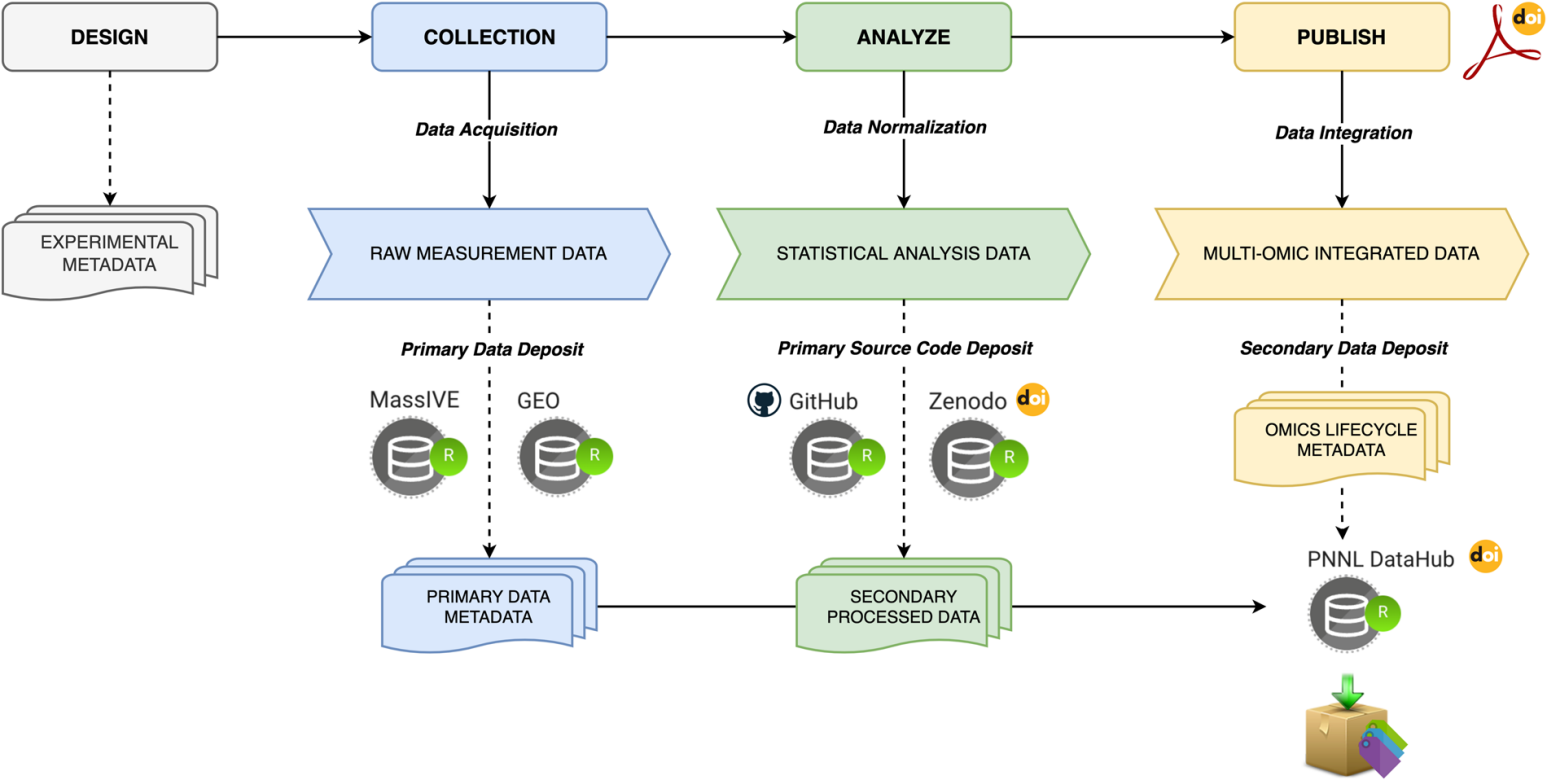
The life cycle of all data generated in OMICs-LHV experiments is detailed. Experimental metadata, raw multi-omics data, and statistically processed datasets are available on PNNL DataHub.

Additionally, links to the raw datasets at public repositories, along with all primary experimental metadata and statistically processed datasets associated with a given experiment can be accessed from a single location search via the PNNL DataHub directory^73^, at each virus specific project landing page^74^, listing all experiments performed with each virus (influenza^75^, Ebola^76^, MERS-CoV^77^, or WNV^78^) or interferon treatment^79^, or from individual dataset DOI landing pages associated with each experiment (45 experiments in total) (Fig. 5). The PNNL DataHub directory pages for each experiment are as follows:

- ***Interferon treatment:*** IFNaCL001^80^, IFNaHUH001^81^, IFNaIHH001^82^, IFNFB001^83^, and IFNMVE001^84^
- ***Influenza:*** ICL102^85^, ICL103^86^, ICL104^87^, ICL105^88^, ICL106^89^, IM101^36^, IM102^90^, and IM103^91^
- ***Ebola:*** EH001^92^, EHUH001^93^, EHUH002^94^, EHUH003^95^, EHUVEC001^96^, EU937001^97^
- ***MERS-CoV:*** MCL001^98^, MCL002^99^, MCL003^100^, MCL004^101^, MCL005^102^, MFB001^103^, MFB002^104^, MFB003^105^, MHAE001^106^, MHAE002^107^, MHAE003^108^, MMVE001^109^, MMVE002^110^, MMVE003^111^, MM001^112^
- ***WNV:*** WDC010^113^, WDC011^114^, WCN002^115^, WCN003^116^, WGCN002^117^, WGCN003^118^, WCB001^119^, WCT001^120^, WLN002^121^, WLN003^122^, WSE001^123^

### Primary experimental metadata

Primary experimental metadata are the structured metadata, in Excel file format, describing the experimental design, data outputs, and phenotypic data associated with each experiment. A brief description of each primary experimental metadata file follows below, and a more detailed description of the contents of the primary experimental metadata files is provided in Supplementary Table 4. All primary experimental metadata files may be accessed on the PNNL DataHub directory pages for each experiment, which are referenced above.

#### Experimental design

The “Experimental Design” document provides an overview of each experiment, including the purpose (*i.e*., what type of multi-omics samples were collected), the model system (cell type, mouse strain and tissue types, or human samples), viruses or treatments and the dosages used, time points at which samples were collected, the number of replicates for each infection and time point condition, and the type(s) of available phenotypic data. Other notes are included as needed. Experimental Design documents are provided on the DataHub directory for all 45 experiments listed above.

#### Dataset summary

The “Dataset Summary” document provides a list of all samples collected in an experiment and the type(s) of analyses that were performed with each sample (*i.e*., the data outputs). Dataset Summary documents are provided on the DataHub directory for all 45 experiments listed above.

#### Workbook

The “Workbook” document provides the results of any phenotypic data collection that was performed for an experiment (note that Workbook files may contain multiple tabs dedicated to different types of phenotypic data). Workbook documents are provided on the DataHub directory for all experiments for which phenotypic data is available.

### Transcriptomics (NCBI BioProject, GEO)

Raw transcriptomics datasets derived from Agilent mRNA or microRNA gene expression arrays were deposited in NCBI GEO under the GEO SuperSeries GSE65575^124^. The collection comprises 60 total GEO Series collections: 2,555 total sample data files (1,162 associated with human cells and 894 associated with mouse cells or tissues). Each dataset, including mRNA and microRNA datasets originating from the same experiment, has its own unique GEO Series accession identifier, except for a few GEO series where both mRNA and miRNA were grouped for publication. Data deposited at the NCBI GEO database comprise transcriptomic metadata, SOFT, MINiML, and TAR/TXT formats with raw probe intensities.

**Fig. 6:**
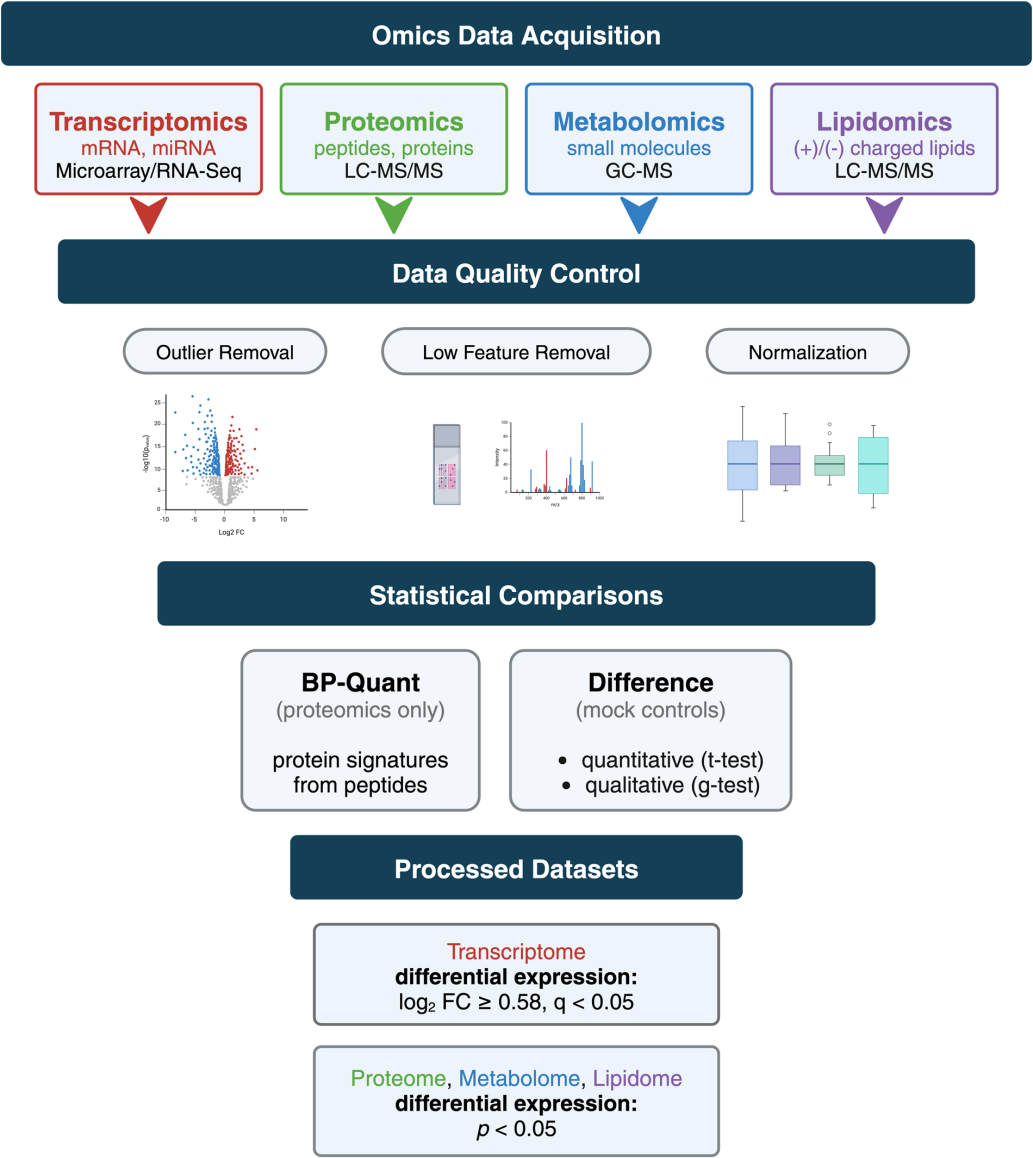
A basic overview of the statistical validation workflow is provided All raw multi-omics datasets were subjected to quality control analyses to remove outliers, eliminate features with low data, and normalize the data. Subsequently, statistical comparisons were made. Statistically processed datasets are available for each experiment in the data records on PNNL DataHub.

Processed transcriptomics datasets, which are more accessible to those without expertise in statistical analyses, also are available in Excel file format on the PNNL DataHub directory (an overview of statistical processing is provided in Fig. 6). For each dataset, the processed data Excel file consists of multiple tabs, including the following: (*i*) A “ReadMe” tab, explaining the contents of the file; (*ii*) A “Normalized_Intensities” tab, containing the normalized, log_2_-transformed intensity values for each probe in each sample; (*iii*) An “Individual_Fold_Changes” tab, containing the log_2_ ratio of each probe in each virus/interferon-treated sample versus the average of the of the time-matched mock-treated controls; (*iv*) A “DE_Test_Results” tab, containing the results of differential expression analyses comparing virus/interferon-treated samples to time-matched mock samples for each probe (this tab includes log_2_ fold-changes, q values, and a flag for statistical significance for each comparison); and (*v*) A “DE_Genes_Only” tab, containing the same information as in the “DE_Test_Results” tab except only for probes that met differential expression criteria. Differential expression criteria are described in the “ReadMe” tabs of all statistically processed dataset Excel files, and further, are described in the Methods section herein (see above).

Below, GEO accessions are provided for each transcriptomics dataset generated under the OMICS-LHV project, along with references to the appropriate data record on the PNNL DataHub, comprising primary experimental metadata and statistically processed datasets.

#### Interferon treatment

- IFNaHUH001 (human Huh 7 cells treated with interferon α and β): mRNA GEO series (24 samples) GSE106522^125^; primary experimental metadata and statistically processed datasets^81^
- IFNaCL001 (human Calu-3 cells treated with interferon α): mRNA GEO series (23 samples) GSE70217^126^; microRNA GEO series (23 samples) GSE70220^127^; primary experimental metadata and statistically processed datasets^80^
- IFNaIHH001 (human IHH cells treated with interferon α): mRNA GEO series (17 samples) GSE71732^128^; microRNA GEO series (16 samples) GSE71733^129^; primary experimental metadata and statistically processed datasets^82^
- IFNFB001 (primary human lung fibroblasts treated with interferon α and β or interferon γ): mRNA GEO series (36 samples) GSE106523^130^; primary experimental metadata and statistically processed datasets^83^
- IFNMVE001 (primary human lung microvascular endothelial cells treated with interferon α and β or interferon γ): mRNA GEO series (35 samples) GSE106524^131^; primary experimental metadata and statistically processed datasets^84^

#### Influenza

- ICL102 (human Calu-3 cells infected with H7N9 and mutants): mRNA GEO series (61 samples) GSE69026^132^; microRNA GEO series (24 samples) GSE69027^133^; primary experimental metadata and statistically processed datasets^85^
- ICL103 (human Calu-3 infected with H5N1 and mutants): mRNA GEO series (80 samples) GSE76599^134^; microRNA GEO series (27 samples) GSE76600^135^; primary experimental metadata and statistically processed datasets^86^
- ICL104 (human Calu-3 cells infected with pH1N1): mRNA GEO series (57 samples) GSE80697^136^; microRNA GEO series (29 samples) GSE80698^137^; primary experimental metadata and statistically processed datasets^87^
- IM101 (C57BL/6 J mouse lungs infected with pH1N1, H5N1, and H5N1 mutants): mRNA GEO series (67 samples) GSE69945^138^; microRNA GEO series (35 samples) GSE69944^139^; primary experimental metadata and statistically processed datasets^36^
- IM102 (C57BL/6 J mouse lungs infected with H7N9 and mutants): mRNA GEO series (66 samples) GSE68945^140^; microRNA GEO series (23 samples) GSE68946^141^; primary experimental metadata and statistically processed datasets^90^
- IM103-ArrayStar (C57BL/6 J mouse lungs infected with pH1N1, H5N1, or H5N1 mutants): mRNA GEO series (37 samples) GSE71759^142^; microRNA GEO series (14 samples) GSE71760^143^; primary experimental metadata and statistically processed datasets^91^
- IM103-AmbryGenetics (C57BL/6 J mouse lungs infected with pH1N1, H5N1, or H5N1 mutants): mRNA GEO series (37 samples) GSE72008^144^; microRNA GEO series (14 samples) GSE72365^145^; primary experimental metadata and statistically processed datasets^91^

#### Ebola

- EH001 (PBMCs from humans naturally infected with Ebola virus and healthy volunteers): Due to ethical considerations, RNAseq data derived from human subjects are not publicly available.
- EHUH001 (human Huh 7-VP30 cells infected with Ebola-ΔVP30 and mutants): mRNA GEO series (119 samples) GSE80058^146^; microRNA GEO series (78 samples) GSE80059^147^; primary experimental metadata and statistically processed datasets^93^
- EHUH003 (human Huh 7-VP30 cells infected with Ebola-ΔVP30 and mutants): mRNA GEO series (48 samples) GSE86539^148^; microRNA GEO series (23 samples) GSE86533^149^; primary experimental metadata and statistically processed datasets^95^
- EHUVEC001 (HUVEC expressing VP30 and infected with Ebola-ΔVP30 and mutants): mRNA GEO series (75 samples) GSE210189^150^; primary experimental metadata and statistically processed datasets^96^
- EU937001 (human U937 cells infected with Ebola-ΔVP30 and mutants): mRNA GEO series (54 samples) GSE80832^151^; microRNA GEO series (38 samples) GSE80833^152^; primary experimental metadata and statistically processed datasets^97^

#### MERS-CoV

- MCL001 (human Calu-3 2B4 cells infected with icMERS-CoV and mutants): mRNA GEO series (64 samples) GSE65574^153^; microRNA GEO series (31 samples) GSE65574^153^; primary experimental metadata and statistically processed datasets^98^
- MFB001 (human primary lung fibroblasts infected with icMERS-CoV): mRNA GEO series (50 samples) GSE79458^154^; microRNA GEO series (27 samples) GSE79459^155^; primary experimental metadata and statistically processed datasets^103^
- MFB002 (human primary lung fibroblasts infected with icMERS-CoV): mRNA GEO series (50 samples) GSE86528^156^; primary experimental metadata and statistically processed datasets^104^
- MFB003 (human primary lung fibroblasts infected with icMERS-CoV): mRNA GEO series (50 samples) GSE100496^157^; primary experimental metadata and statistically processed datasets^105^
- MHAE001 (human primary airway epithelium infected with icMERS-CoV): mRNA GEO series (50 samples) GSE81909^158^; microRNA GEO series (27 samples) GSE81852^159^; primary experimental metadata and statistically processed datasets^106^
- MHAE002 (human primary airway epithelium infected with icMERS-CoV): mRNA GEO series (50 samples) GSE86530^160^; primary experimental metadata and statistically processed datasets^107^
- MHAE003 (human primary airway epithelium infected with icMERS-CoV): mRNA GEO series (50 samples) GSE100504^161^; primary experimental metadata and statistically processed datasets^108^
- MMVE001 (human primary lung microvascular endothelial cells infected with icMERS-CoV): mRNA GEO series (49 samples) GSE79218^162^; microRNA GEO series (14 samples) GSE79216^163^; primary experimental metadata and statistically processed datasets^109^
- MMVE002 (human primary lung microvascular endothelial cells infected with icMERS-CoV): mRNA GEO series (50 samples) GSE86529^164^; primary experimental metadata and statistically processed datasets^110^
- MMVE003 (human primary lung microvascular endothelial cells infected with icMERS-CoV): mRNA GEO series (50 samples) GSE100509^165^; primary experimental metadata and statistically processed datasets^111^
- MM001 (C57BL/6J-hDPP4 mouse lungs infected with icMERS-CoV MA1): mRNA GEO series (46 samples) GSE108594^166^; primary experimental metadata and statistically processed datasets^112^

#### WNV

- WCN002 (primary mouse cortical neurons infected with WNV and mutant): mRNA GEO series (35 samples) GSE67473^167^; microRNA GEO series (8 samples) GSE67474^168^; primary experimental metadata and statistically processed datasets^115^
- WCN003 (primary mouse cortical neurons infected with WNV and mutant): mRNA GEO series (35 samples) GSE67473^167^; microRNA GEO series (8 samples) GSE67474^168^; primary experimental metadata and statistically processed datasets^116^
- WDC010 (primary mouse dendritic cells infected with WNV and mutant): mRNA GEO series (33 samples) GSE74628^169^; primary experimental metadata and statistically processed datasets^113^
- WDC011 (primary mouse dendritic cells infected with WNV and mutant): mRNA GEO series (33 samples) GSE75222^170^; primary experimental metadata and statistically processed datasets^114^
- WGCN002 (primary mouse granule cell neurons infected with WNV and mutant): mRNA GEO series (35 samples) GSE68380^171^; microRNA GEO series (8 samples) GSE68381^172^; primary experimental metadata and statistically processed datasets^117^
- WGCN003 (primary mouse granule cell neurons infected with WNV and mutant): mRNA GEO series (35 samples) GSE68380^171^; microRNA GEO series (9 samples) GSE68381^172^; primary experimental metadata and statistically processed datasets^118^
- WCB001 (C57BL/6 J mouse cerebellum infected with WNV and mutant): mRNA GEO series (60 samples) GSE77192^173^; microRNA GEO series (30 samples) GSE77160^174^; primary experimental metadata and statistically processed datasets^119^
- WCT001 (C57BL/6 J mouse cortex infected with WNV and mutant): mRNA GEO series (60 samples) GSE77193^175^; microRNA GEO series (30 samples) GSE77161^176^; primary experimental metadata and statistically processed datasets^120^
- WLN002 (C57BL/6 J mouse lymph nodes infected with WNV and mutant): mRNA GEO series (51 samples) GSE78888^177^; microRNA GEO series (26 samples) GSE78887^178^; primary experimental metadata and statistically processed datasets^121^

It is important to note that three experiments performed for the OMICS-LHV project, with raw transcriptomics data deposited in the public domain, have not been discussed herein. These experiments include EIHH001 (mRNA, miRNA; GSE65573^179^) and EIHH002 (mRNA, miRNA; GSE69942^180^, GSE69943^181^), which were excluded due to excessive drift in transcript expression over time in mock-infected samples; and MDC001 (mRNA; GSE79172^182^), which was excluded due to lack of efficient infection.

### Proteomics, metabolomics, and lipidomics (MassIVE)

Raw proteomics, metabolomics, and lipidomics datasets derived from mass spectrometry analyses were deposited in the MassIVE database. The collection comprises >21,000 datasets (12,514 associated human cells and 8,033 associated with mouse cells and/or tissues) and a total of 67 experimental deposits. Each dataset has a unique accession number. Data deposited in the MassIVE database comprise GC-MS data (metabolites); LC-MS/MS data (lipids); and mass spectrometry data corresponding to instrument files, mzML, and MSGF + MS/MS search for peptide identifications used to populate AMT tag databases (proteomics).

Processed proteomics, metabolomics, and lipidomics datasets, which are more accessible to those without expertise in statistical analyses, also are available in Excel file format (an overview of statistical processing is provided in Fig. 6). For each dataset, the processed data Excel file consists of multiple tabs, including the following: (*i*) A “ReadMe” tab, explaining the contents of the file; (*ii*) A “Normalized_Data” tab, containing the normalized, log_2_-transformed values for each feature in each sample; (*iii*) A “DA_Test_Results” tab, containing the results of differential abundance analyses comparing virus/interferon-treated samples to time-matched mock samples for each feature (this tab includes log_2_ fold-changes, *p* values, and a flag for statistical significance for each comparison); and (*v*) A “DA_Proteins/Metabolites/Lipids_Only” tab, containing the same information as in the “DA_Test_Results” tab except only for features that met differential abundance criteria. Differential abundance criteria are described in the “ReadMe” tabs of all statistically processed dataset Excel files, and further, are described in the Methods section herein (see above).

Below, MassIVE accessions are provided for each dataset generated under the OMICS-LHV project, along with references to the appropriate data record on the PNNL DataHub.

#### Influenza

- ICL102 (human Calu-3 cells infected with H7N9 and mutants): proteomics MSV000079164^183^; metabolomics MSV000079156^184^; lipidomics MSV000079386^185^; primary experimental metadata and statistically processed datasets^85^
- ICL103 (human Calu-3 cells infected with H5N1 and mutants): proteomics MSV000079459^186^; metabolomics MSV000079460^187^; lipidomics MSV000080023^188^; primary experimental metadata and statistically processed datasets^86^
- ICL104 (human Calu-3 cells infected with pH1N1): proteomics MSV000080026^189^; metabolomics MSV000079704^190^; lipidomics MSV000081049^191^; primary experimental metadata and statistically processed datasets^87^
- IM102 (C57BL/6 J mouse lungs infected with H7N9 and mutants): proteomics MSV000079343^192^; metabolomics MSV000079206^193^; lipidomics MSV000079542^194^ and MSV000089784^195^; primary experimental metadata and statistically processed datasets^90^
- IM103 (C57BL/6 J mouse lungs infected with pH1N1, H5N1, and H5N1 mutants): proteomics MSV000079469^196^; metabolomics MSV000079327^197^; lipidomics MSV000080027^198^; primary experimental metadata and statistically processed datasets^91^

#### Ebola

- EH001 (plasma from humans naturally infected with Ebola virus): proteomics MSV000080129^199^; metabolomics MSV000080129^199^; lipidomics MSV000080129^199^; primary experimental metadata and statistically processed datasets^92^
- EHUH002 (human Huh 7-VP30 cells infected with Ebola-ΔVP30 and mutants): proteomics MSV000081041^200^; metabolomics MSV000081042^201^; lipidomics MSV000081892^202^; primary experimental metadata and statistically processed datasets^94^

#### MERS-CoV

- MCL002 (human Calu-3 2B4 cells infected with icMERS-CoV and mutants): proteomics MSV000080025^203^; metabolomics MSV000080022^204^; lipidomics MSV000081045^205^; primary experimental metadata and statistically processed datasets^99^
- MCL003 (human Calu-3 2B4 cells infected with icMERS-CoV): proteomics MSV000079152^206^; metabolomics MSV000079153^207^; lipidomics MSV000079154^208^; primary experimental metadata and statistically processed datasets^100^
- MFB001 (human primary lung fibroblasts infected with icMERS-CoV): proteomics MSV000079701^209^; metabolomics MSV000079700^210^; lipidomics MSV000081046^211^; primary experimental metadata and statistically processed datasets^103^
- MFB002 (human primary lung fibroblasts infected with icMERS-CoV): proteomics MSV000080028^212^; metabolomics MSV000080018^213^; lipidomics MSV000081050^214^; primary experimental metadata and statistically processed datasets^104^
- MFB003 (human primary lung fibroblasts infected with icMERS-CoV): proteomics MSV000081887^215^; metabolomics MSV000081043^216^; lipidomics MSV000081893^217^; primary experimental metadata and statistically processed datasets^105^
- MHAE001 (human primary airway epithelium infected with icMERS-CoV): proteomics MSV000083529^218^; metabolomics MSV000081889^219^; lipidomics MSV000083533^220^; primary experimental metadata and statistically processed datasets^106^
- MHAE002 (human primary airway epithelium infected with icMERS-CoV): proteomics MSV000083530^221^; metabolomics MSV000081890^222^; lipidomics MSV000083534^223^; primary experimental metadata and statistically processed datasets^107^
- MHAE003 (human primary airway epithelium infected with icMERS-CoV): proteomics MSV000083531^224^; metabolomics MSV000081891^225^; lipidomics MSV000083535^226^; primary experimental metadata and statistically processed datasets^108^
- MMVE001 (human primary lung microvascular endothelial cells infected with icMERS-CoV): proteomics MSV000079703^227^; metabolomics MSV000079702^228^; lipidomics MSV000081047^229^; primary experimental metadata and statistically processed datasets^109^
- MMVE002 (human primary lung microvascular endothelial cells infected with icMERS-CoV): proteomics MSV000080029^230^; metabolomics MSV000080017^231^; lipidomics MSV000081051^232^; primary experimental metadata and statistically processed datasets^110^
- MMVE003 (human primary lung microvascular endothelial cells infected with icMERS-CoV): proteomics MSV000081888^233^; metabolomics MSV000081044^234^; lipidomics MSV000081894^235^; primary experimental metadata and statistically processed datasets^111^
- MM001 (C57BL/6J-hDPP4 mouse lungs infected with icMERS-CoV MA1): proteomics MSV000083532^236^; metabolomics MSV000083537^237^; lipidomics MSV000083536^238^; primary experimental metadata and statistically processed datasets^112^

#### WNV

- WCB001 (C57BL/6 J mouse cerebellum infected with WNV and mutant): proteomics MSV000080360^239^; metabolomics MSV000080020^240^; lipidomics MSV000081052^241^; primary experimental metadata and statistically processed datasets^119^
- WCT001 (C57BL/6 J mouse cortex infected with WNV and mutant): proteomics MSV000080361^242^; metabolomics MSV000080019^243^; lipidomics MSV000081048^244^; primary experimental metadata and statistically processed datasets^120^
- WLN003 (C57BL/6 J mouse lymph nodes infected with WNV and mutant): proteomics MSV000080024^245^; metabolomics MSV000080021^246^; lipidomics MSV000080354^247^; primary experimental metadata and statistically processed datasets^122^
- WSE001 (antibiotics-treated C57BL/6 J mouse serum infected with WNV): proteomics MSV000091193^248^; metabolomics MSV000091199^249^; primary experimental metadata and statistically processed datasets^123^

### ChIP-Seq and MeDIP-Seq (NCBI BioProject, GEO)

Raw ChIP-Seq and MeDIP-Seq datasets were deposited in NCBI GEO as described above. ChIP-Seq datasets (derived from experiments ICL105 and MCL004) and MeDIP-Seq datasets (derived from experiments ICL106 and MCL005) are available under GEO accessions GSE108881^250^ and GSE108882^251^, respectively. Data records are available on the PNNL DataHub.

## Technical Validation

### Assessment of infection status

For infection experiments in cell cultures, viral replication was confirmed by quantification of infectious virus particles in the cell culture medium for the same monolayers used for multi-omics analyses and/or by quantification of virus infectivity (*i.e*., percent infection) for cultures prepared in parallel. For infection experiments in mice, viral infection and replication was confirmed by quantification of infectious virus particles in the appropriate target tissue type. For the study of humans naturally infected with Ebola virus, all Ebola virus-positive individuals had an Ebola virus-positive RT-PCR test prior to admission to an Ebola Treatment Center and recruitment to our study. In addition, we confirmed the presence and quantity of Ebola virus genomes in isolated PBMCs using quantitative RT-PCR. Viral titers, infectivity data, and Ebola virus loads in human patients are available in the primary experimental metadata workbook documents provided with each data record on DataHub.

### Assessment of Disease/Phenotype

For infection experiments in cell cultures, cell monolayers were observed by visual inspection for the development of cytopathic effects at each sample collection time point. For influenza and MERS-CoV infections in mice, body weights and survival were monitored daily. As expected, all mice infected with wild-type viruses exhibited body weight loss and survival rates consistent with previous experiments at the given inoculation dosages. Weight loss and survival data are available in the primary experimental metadata workbook documents provided with each data record on DataHub. Since body weight loss was not expected in mice infected with WNV, no body weights were collected for WNV experiments. For humans naturally infected with Ebola virus, information on the onset of disease and type of disease (wet versus dry) at hospital admission are available in the primary experimental metadata workbook document on DataHub.

### Sample tracking and data randomization

Experiment and sample naming schemes were developed at the beginning of the program to capture treatment, host, cell/tissue type and replicate number in a consistent manner. Template documents were created to capture experimental design, metadata, and sample inventories for each experiment. These documents are included with our public data downloads. Prior to processing, all samples within an experiment were randomized, balanced, and blocked based on the mass spectrometry capability being utilized and throughput of each instrument (e.g., plates, columns, day). The randomization method accounted for experimental design and confounding factors introduced from sample handling and instrument processing to assure appropriate estimates of Type 1 and 2 error rates are attained.

### Statistical evaluation of experimental variation and data errors

In the analysis of differential molecular intensities (abundance measures), poor quality data can bias downstream statistical analyses and biological interpretation. For microarray data, array outliers were detected based on three criteria: abnormal distribution of probe intensities, abnormal expression profile, or abnormal clustering behavior. Box plots of raw, background-corrected and normalized intensities are used to identify samples for which correction methods do not fix problems intensity distribution. Expression profiles and clustering of individual arrays based on the normalized data are used to identify individual arrays that are vastly skewed from other technical replicates within a treatment group. When the weight of evidence from all three outlier methods indicates that one or more arrays are outliers, they were removed from the analysis and the normalization and outlier steps are repeated. The process was repeated until no more outliers were found. For all MS data, we developed a novel multivariate statistical strategy for the identification of individual MS runs with extreme abundance distributions^65^. We first summarize each run with 5 metrics: correlation with other runs in the same experiment, fraction of missing data, median absolute deviation of identified molecules within a run, skew, and kurtosis. We then employ a robust principle component analysis (rPCA) algorithm^252^ based on project-pursuit to estimate the eigenvalues, and subsequent scores obtained from the projections of the metrics on the eigenvectors. This allows us to obtain a robust estimate of the covariance matrix and calculate robust Mahalanobis distance (rMd), the distance of an individual MS run from the center of the data. The rMd squared values associated with the molecular abundance vector is used as a score to assess whether individual runs are outliers within a given experiment defined by a large rMd-PAV score such that the calculated squared distance exceeds a critical value of the chi-squared distributed with q degrees of freedom (χ_q_^2^) distribution specified a priori.

## Usage Notes

**Fig. 7:**
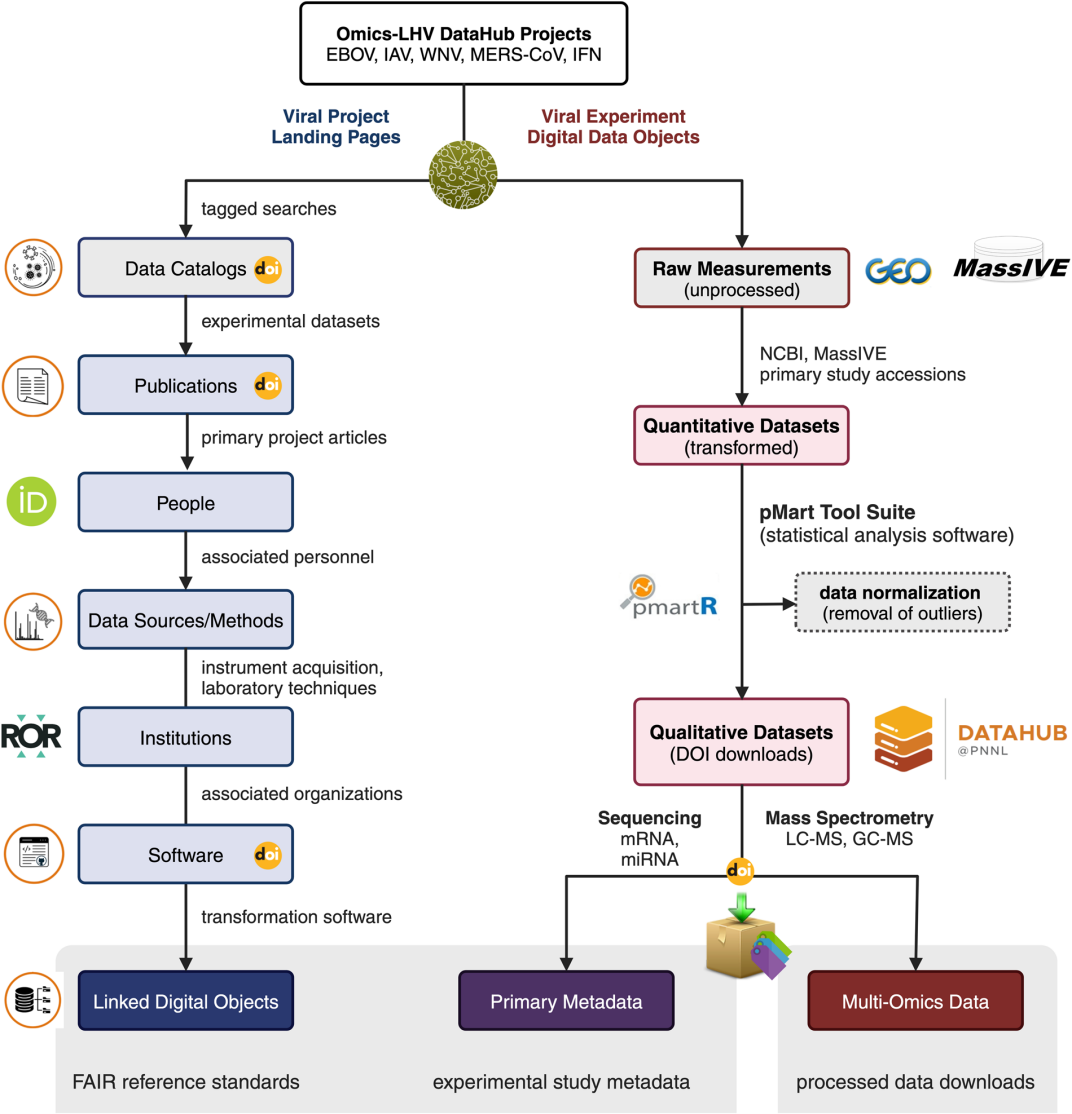
Graphical overview of the OMICS-LHV multi-omics project collections and processed digital data downloads made available from PNNL DataHub. The PNNL DataHub OMICS-LHV project pages contain comprehensive data catalog information (left) and processed dataset package DOI downloads (right), linked to necessary context resource information supporting experimental and computational methods and relationships. EBOV, Ebola virus; IAV, influenza A virus; WNV, West Nile virus; MERS-CoV, Middle East respiratory Syndrome-related coronavirus; IFN, interferon.

As described above, all data records (including links to raw multi-omics datasets on public repositories, along with the associated primary experimental metadata and statistically processed datasets) can be accessed from a single location, via the Pacific Northwest National Laboratory DataHub repository^73^. Through the PNNL DataHub, users can browse OMICS-LHV data records and other project catalogues, as well as publications, data sources (applied instrument capabilities), software (related source code required for reproducibility), and people (project associated personal) assigned to each dataset or PNNL DataHub landing page (Fig. 7). Data record pages were created in PNNL DataHub for each experiment (45 in total). On each data record page, links to raw multi-omics data deposited in public repositories were provided and primary experimental metadata and statistically processed multi-omics datasets were uploaded. Each data record was assigned a unique and persistent DOI registered through the U.S. Department of Energy Office of Scientific and Technical Information (OSTI), a Department of Energy registry provider leveraging linked open research services with Crossref, DataCite, and ORCID. All DOI registration metadata through OSTI are permanently preserved for sustainable project recordkeeping and contain all corresponding primary database accession submissions at domain community repositories with primary raw data publications where applicable. A comprehensive list of all sample types, data types, data sources, and software tools linked to processed dataset downloads can be referenced from Supplementary Table 5. Data records can be accessed via the PNNL DataHub under the main project landing page^73^, via a subset of virus-specific project landing pages^75–79^, or by directly accessing a data record for an individual experiment (links to individual experiments are referenced in the Data Records section, above). All data associated with each data record, including “READ ME” files describing cut-off criteria for statistically processed multi-omics datasets, may be downloaded from each data record page. DOI data and metadata download contents contain machine-actionable file formats (.txt, .csv, .json, etc.) required for discovery and reuse.

In efforts to enable dataset citation discovery, transparency, and reproducibility, we ask that the following data citation policy reported here be applied for all “NIAID Modeling Host Responses to Understand Severe Human Virus Infections, Multi-Omic Viral Dataset Catalog Collection” project digital data assets listed at the PNNL DataHub institutional repository and corresponding domain database accessions. Referencing and reuse of linked NIH-funded project processed datasets, raw measurement datasets, and related metadata download materials acknowledge all primary and secondary dataset citations where applicable and direct corresponding journal articles (in reference to grant # **U19AI106772**) where allowable. All digital data DOI downloads have been provided a CC BY 4.0 license and a CC0 1.0 license. At the PNNL DataHub institutional repository project pages, we ask that users please cite each individual dataset DOI provided at the download page and any corresponding journal article publications for reuse. Reference citations, where applicable, should provide the necessary metadata information and direct data repository citations required to support, corroborate, verify, and otherwise determine the legitimacy of the research findings provided (data and code) from scholarly publications and corresponding project data releases.

### Supplementary information

Supplementary Table 1

Supplementary Table 2

Supplementary Table 3

Supplementary Table 4

Supplementary Table 5

Human Data Submission Checklist

## Data Availability

Reported multi-omics data processing and analysis software pmartR and small molecule identification and annotation software LIQUID have been made publicly available and are openly accessible to the global scientific community. These software packages can be formally cited from their corresponding Zenodo citations^253,254^. All GitHub source code repositories, used to verify and corroborate data source code collections, have been assigned corresponding globally unique and persistent DOI at Zenodo under a CC BY 4.0 licence. Potential users should consult corresponding GitHub landing pages for any additional licences, references, and disclaimers provided.
